# Adipose tissue protects against sepsis-induced muscle weakness in mice: from lipolysis to ketones

**DOI:** 10.1186/s13054-019-2506-6

**Published:** 2019-07-01

**Authors:** Chloë Goossens, Ruben Weckx, Sarah Derde, Thomas Dufour, Sarah Vander Perre, Lies Pauwels, Steven E. Thiessen, Paul P. Van Veldhoven, Greet Van den Berghe, Lies Langouche

**Affiliations:** 1 Clinical Division and Laboratory of Intensive Care Medicine, Department of Cellular and Molecular Medicine, KU Leuven, 3000 Leuven, Belgium; 2 Laboratory for Lipid Biochemistry and Protein Interactions, Department of Cellular and Molecular Medicine, KU Leuven, 3000 Leuven, Belgium

**Keywords:** Adipose tissue, Skeletal muscle, Sepsis, Metabolism, Muscle weakness

## Abstract

**Background:**

ICU-acquired weakness is a debilitating consequence of prolonged critical illness that is associated with poor outcome. Recently, premorbid obesity has been shown to protect against such illness-induced muscle wasting and weakness. Here, we hypothesized that this protection was due to increased lipid and ketone availability.

**Methods:**

In a centrally catheterized, fluid-resuscitated, antibiotic-treated mouse model of prolonged sepsis, we compared markers of lipolysis and fatty acid oxidation in lean and obese septic mice (*n* = 117). Next, we compared markers of muscle wasting and weakness in septic obese wild-type and adipose tissue-specific ATGL knockout (AAKO) mice (*n* = 73), in lean septic mice receiving either intravenous infusion of lipids or standard parenteral nutrition (PN) (*n* = 70), and in lean septic mice receiving standard PN supplemented with either the ketone body 3-hydroxybutyrate or isocaloric glucose (*n* = 49).

**Results:**

Obese septic mice had more pronounced lipolysis (*p* ≤ 0.05), peripheral fatty acid oxidation (*p* ≤ 0.05), and ketogenesis (*p* ≤ 0.05) than lean mice. Blocking lipolysis in obese septic mice caused severely reduced muscle mass (32% loss vs. 15% in wild-type, *p* < 0.001) and specific maximal muscle force (59% loss vs. 0% in wild-type; *p* < 0.001). In contrast, intravenous infusion of lipids in lean septic mice maintained specific maximal muscle force up to healthy control levels (*p* = 0.6), whereas this was reduced with 28% in septic mice receiving standard PN (*p* = 0.006). Muscle mass was evenly reduced with 29% in both lean septic groups (*p* < 0.001). Lipid administration enhanced fatty acid oxidation (*p* ≤ 0.05) and ketogenesis (*p* < 0.001), but caused unfavorable liver steatosis (*p* = 0.01) and a deranged lipid profile (*p* ≤ 0.01). Supplementation of standard PN with 3-hydroxybutyrate also attenuated specific maximal muscle force up to healthy control levels (*p* = 0.1), but loss of muscle mass could not be prevented (25% loss in both septic groups; *p* < 0.001). Importantly, this intervention improved muscle regeneration markers (*p* ≤ 0.05) without the unfavorable side effects seen with lipid infusion.

**Conclusions:**

Obesity-induced muscle protection during sepsis is partly mediated by elevated mobilization and metabolism of endogenous fatty acids. Furthermore, increased availability of ketone bodies, either through ketogenesis or through parenteral infusion, appears to protect against sepsis-induced muscle weakness also in the lean.

**Electronic supplementary material:**

The online version of this article (10.1186/s13054-019-2506-6) contains supplementary material, which is available to authorized users.

## Background

Critically ill patients frequently develop weakness of limb and respiratory muscles. Such intensive care unit (ICU)-acquired weakness has a high prevalence and is associated with greater post-ICU impairment, prolonged hospitalization, delayed rehabilitation, and late death [1, 2]. Particularly patients suffering from sepsis are at risk of developing ICU-acquired weakness [3].

Profound loss of muscle mass and quality characterizes ICU-acquired weakness [4]. This loss of muscle mass is caused by activated myofibrillary breakdown without compensatory protein synthesis [5–8]. In addition, the loss of myofiber quality is related to insufficiently activated autophagy and ongoing inflammation [9–11]. Muscle regeneration is also severely impaired in prolonged critically ill patients, which hampers rehabilitation [12]. Several interventions, such as aggressive sepsis treatment, early mobilization, prevention of hyperglycemia, and withholding early parenteral nutrition (PN), have been shown to partially protect against ICU-acquired weakness [4, 9, 13, 14]. Nevertheless, to reduce the prevalence of this debilitating condition, additional clinical interventions are still required.

Recently, premorbid overweight/obesity has been shown to protect against muscle wasting and weakness in both critically ill patients and in septic mice [15]. In these septic mice, the overweight/obese preserved their muscle mass while losing fat mass. In contrast, lean septic mice prioritized the maintenance of fat mass over muscle mass [15]. Markers of fatty acid metabolism and ketogenesis also appeared more increased in overweight/obese than in lean septic mice [15]. This suggested an intrinsically different metabolic response to sepsis with overweight/obesity. Importantly, diet-induced obesity does typically enhance basal lipolysis, hepatic lipid metabolism, fatty acid oxidation, and ketogenesis [16–23].

Such an altered metabolic response in obese individuals, with increased release and metabolism of fatty acids to ketones, might mediate the observed protection against sepsis-induced muscle wasting and weakness. Indeed, in patients suffering from pancreatic cancer cachexia, a high-fat, ketogenic diet protected against muscle wasting. Additionally, in elite athletes, muscle function improved after exogenous ketone body administration [24, 25]. Ketone bodies exert several functions that could benefit the muscle during critical illness. As energy substrates, they can alter muscle substrate metabolism from the use of glycogen to 3-hydroxybutyrate (3-HB) [25–27]. Furthermore, as signaling molecules, ketone bodies have anti-inflammatory and autophagy-stimulating actions and can induce mTOR-mediated protein synthesis and muscle regeneration [28–32].

As a general hypothesis, we state that the protection against ICU-acquired weakness in overweight/obese critically ill patients can be explained by their enhanced ability to release and metabolize fatty acids from their excess adipose tissue. This hypothesis was tested with four consecutive studies in a mouse model of prolonged abdominal sepsis [33]. Of note, this centrally catheterized, fluid-resuscitated, and antibiotic-treated mouse model results in muscle wasting and weakness that is comparable to ICU-acquired weakness in patients [15]. We aimed to assess (1) whether overweight/obesity causes enhanced mobilization and metabolism of endogenous fatty acids during sepsis and (2) whether such altered metabolic response protects the skeletal muscle. Furthermore, we aimed to evaluate (3) whether the obesity-induced muscle protection can be mimicked in lean septic mice by increased lipid availability. Lastly, we aimed to investigate (4) whether the observed muscle protection is mediated by ketone bodies that either function as alternative energy substrates or as signaling molecules.

## Methods

### Animal study design

Male, 24-week-old mice were anesthetized and a catheter was placed in the central jugular vein, followed by cecal ligation and puncture to induce sepsis [33, 34]. Unless indicated, 57BL/6JRj mice (Janvier SAS, Chassal, France) were used. After surgery, mice were fasted and received intravenous fluid resuscitation for 20 h. Fasting was done to mimic the clinical setting in which PN is initially withheld. From day 1 onward, mice received standard mixed PN (5.8 kcal/day; Olimel N7E, Baxter, Lessines, Belgium) unless indicated. Throughout the study, mice were given antibiotics and pain medication. Pain/discomfort was assessed twice daily based on the Mouse Grimace Score [35], and cumulative illness scores were calculated to assess the severity of illness. Individually caged healthy mice receiving standard chow at the same daily caloric intake as septic mice (pair-fed) were used as controls.

#### Study 1 - Fatty acid mobilization and metabolism in lean and overweight/obese septic mice (*n*=117)

After 12 weeks on standard chow (10% fat, E15745-04, ssniff, Soest, Germany) or a high-fat diet (45% fat, E15744-34, ssniff), lean and overweight/obese mice were randomized to “healthy control” or “sepsis”. They were sacrificed after either 1 or 5 days [day 1: lean healthy control *n*=15, obese healthy control *n*=10, lean sepsis *n*=15, obese sepsis *n*=15; day 5: lean healthy control *n*=17, obese healthy control *n*=15, lean sepsis *n*=15, obese sepsis *n*=15].

#### Study 2 – Effect of blocking fatty acid mobilization in overweight/obese septic mice on muscle wasting and weakness (*n*=73)

Adipose triglyceride lipase (ATGL)-flox mice were bred to Adipoq-Cre-mice to generate adipose tissue-specific ATGL knockout (AAKO; ATGL^flox/flox^ Cre/+) and wild-type (ATGL^flox/flox^ +/+) mice (Additional file 5). After 12 weeks on a high-fat diet, overweight/obese AAKO and wild-type mice were randomized to “healthy control” or “sepsis” and sacrificed after 5 days [wild-type healthy control *n*=19, AAKO healthy control *n*=18, wild-type sepsis *n*=19, AAKO sepsis *n*=17].

#### Study 3 – Effect of increased lipid availability on muscle wasting and weakness in lean septic mice (*n*=70)

From day 1 of sepsis onward, lean septic mice randomly received isocaloric amounts of either standard mixed PN (Olimel N7E: 35% lipids, 49% glucose and 16% amino acids), or a lipid-rich PN consisting of mainly long- and medium-chain triglycerides (5.8 kcal/day obtained from: 90% lipids (Smoflipid® Lipid Injectable Emulsion, Fresenius Kabi, Schelle, Belgium, containing 30% soybean oil, 30% medium-chain triglycerides, 15% fish oil and 25% olive oil) and 10% glucose). Mice were sacrificed after 5 days [healthy control *n*=24, sepsis receiving PN *n*=23, sepsis receiving lipid-rich PN *n*=23].

#### Study 4 – Effect of increased ketone body availability on muscle wasting and weakness in lean septic mice (*n*=49)

From day 1 of sepsis onward, lean septic mice received standard mixed PN supplemented with twice-daily subcutaneous bolus injections of isocaloric and isovolumetric amounts of either D-glucose (6.25 mg/g/day; PN+gluc) or D,L-3-HB sodium salt (5 mg/g/day; PN+3-HB). Mice were sacrificed after 5 days [healthy control *n*=15, sepsis PN+gluc *n*=17, sepsis PN+3-HB *n*=17].

All animals were treated according to the Principles of Laboratory Animal Care (U.S. national Society for Medical Research) and the Guide for Care and Use of Laboratory Animals (National Institutes of Health). The Institutional Ethical Committee for Animal Experimentation of the KU Leuven had approved the protocols for these animal studies (project numbers P50/2015 and P009/2016). Mice in study 1 were sacrificed after 1 or 5 days, to assess both the acute and prolonged lipolytic response to sepsis (plasma and ex vivo glycerol) [36, 37]. Mice in study 2-4 were sacrificed after 5 days, the timeframe required to develop sepsis-induced muscle weakness [15]. Assessment of muscle weakness by ex vivo muscle force measurements was the primary endpoint of these studies. Data on survival are provided in Table 1. Additional information is provided in Additional file 7.

**Table 1:** Survival until day 5 of septic mice in the different mouse cohorts

	Randomization	5-day survival (number, %)	*p* value
Study 1: Fatty acid mobilization and metabolism in lean and obese septic mice	Lean	15/18 (83)	1.0
	Obese	15/18 (83)	
Study 2: Effect of blocking fatty acid mobilization in obese septic mice on the muscle	Wild-type	19/22 (86)	0.1
	AAKO	17/25 (68)	
Study 3: Effect of increased lipid availability on the muscle in lean septic mice	PN	23/25 (92)	0.8
	Lipid PN	23/26 (88)	
Study 4: Effect of increased ketone body availability on the muscle in lean septic mice	PN+gluc	17/20 (85)	0.8
	PN+3-HB	17/21 (81)	

In all healthy control groups and day 1 septic mice, survival was 100%. [*AAKO* adipose tissue-specific adipose triglyceride lipase knockout, *PN* parenteral nutrition, *Lipid PN* parenteral nutrition consisting mainly of long- and medium-chain triglycerides, *PN+gluc* mice receiving PN supplemented with glucose, *PN+3-HB* mice receiving PN supplemented with ketone body 3-hydroxybutyrate]

### Ex vivo muscle force

Directly after euthanasia, the extensor digitorum longus (EDL) muscle was carefully dissected and suspended in a temperature controlled (30 °C) organ bath filled with HEPES-fortified Krebs-Ringer solution to measure muscle force (300C-LR Dual-Mode muscle lever, Aurora Scientific, Ontario, Canada). The small size of the EDL guaranteed proper diffusion of oxygen during the procedure. Maximal isometric tetanic force of the EDL muscle was measured by averaging three consecutive tetanic stimuli (180 Hz stimulation frequency, 200 ms duration, 0.2 ms pulse width, 2 min rest intervals). Specific maximal isometric tetanic force was calculated by dividing the maximal isometric tetanic force with the muscle cross-sectional area. Additional information is provided in Additional file 7.

### Tissue analyses

For practical reasons, tibialis anterior (TA) muscle was used for histology and muscle mass assessment, whereas the larger gastrocnemius muscle was homogenized and used for gene, protein, and metabolite analyses.

#### Tissue composition

To eliminate potential bias from illness- or resuscitation-related changes in fluid content, dry weight of isolated tissues was obtained by a freeze-drying process. Triglyceride and glycogen content was measured with commercially available kits (triglyceride quantification kit Ab65336, glycogen assay kit Ab65620, Abcam, Cambridge, UK).

#### Lipolysis

Glycerol release was assessed in epididymal adipose tissue explants with a commercially available kit (Glycerol Assay Kit MAK117, Sigma-Aldrich, Saint Louis, MO, USA).

#### Gene expression

Messenger RNA was isolated, and cDNA was quantified in real time as previously documented [38]. Commercial TaqMan® assays (Applied Biosystems, Carlsbad, CA, USA) were used for all gene expression analyses (Additional file 6: Table S1). Data were normalized to *Rn18s* or *Hprt* and expressed as fold change of the mean of controls.

#### Protein expression analyses

Protein isolation was executed as previously described [39]. Immunoblotting was performed with primary antibodies (Additional files 1, 2, 3, 4, 5, and 6) and secondary horseradish peroxidase-conjugated antibodies. Blots were visualized with the G:BOX Chemi XRQ (SynGene, Bangalore, India) and analyzed with SynGene software. Data were normalized to β-actin levels and expressed as fold change of the mean of controls.

#### Histology

Cross-sectional paraffin sections of TA muscle were stained with a monoclonal antibody against PAX7 (1/100, Mab1675, R&D Systems, Minneapolis, MN, USA, RRID:AB_2159833) and a HRP-linked secondary antibody (Additional files 1, 2, 3, 4, 5, and 6). Histological scoring of liver steatosis was performed on hematoxylin and eosin-stained liver sections.

#### Palmitate oxidation

Oxidation of [1-^14^C]-labeled fatty acids was measured in tissue homogenates using a HEPES-fortified modified Krebs-Henseleit buffer [40]. Oxidation rates are expressed as nmol produced [^14^C]CO_2_ and [^14^C]-labeled acid-soluble metabolites per gram wet weight per minute. Additional information is provided in Additional file 7.

### Plasma analyses

Plasma glucose concentrations were measured in whole blood with a glucose meter after cardiac puncture (Accu-check, Roche, Basel, Switzerland). Plasma glycerol, TNF-α, LDL, HDL, triglycerides, free fatty acids, insulin, and 3-HB were measured using commercially available kits: Glycerol Assay Kit (MAK117, Sigma-Aldrich), Mouse TNF-alpha Quantikine HS ELISA Kit (MHSTA50; R&D Systems), LDL-Cholesterol assay (DZ128A-K; Diazyme Laboratories, Poway, CA, USA), HDL-Cholesterol assay (DZ129A-K; Diazyme), triglyceride quantification kit (Ab65336; Abcam), Free Fatty Acid Fluorometric Assay Kit (7010310; Cayman Chemical Company, Ann Arbor, MI, USA), Insulin Mouse Ultra Sensitive ELISA (90080, Crystal Chem, Downers Grove, IL, USA), and EnzyChrom™ Ketone Body Assay Kit (EKBD-100; Bioassay Systems, Hayward, CA, USA).

### Statistics

Normally distributed data were compared with one-way analysis of variance (ANOVA) with post hoc Fisher’s LSD test (Student’s *t* test) for multiple comparisons, where necessary, after log- or (double) square root-transformation to obtain a near-normal distribution (JMP® Pro 12, SAS Institute Inc., Cary, NC, USA). Not-normally distributed data were analyzed with non-parametric Wilcoxon tests. Two-sided *p* values ≤ 0.05 (α-level of 5%) were considered statistically significant in all analyses. Data are presented as bars with whiskers, showing means and standard error of the mean (SEM). Post hoc *p* values are plotted on the figures as § *p* ≤ 0.05, §§ *p* ≤ 0.01, §§§ *p* ≤ 0.001, between septic and healthy control mice, and * *p* ≤ 0.05, ** *p* ≤ 0.01, *** *p* ≤ 0.001 between groups of septic mice.

## Results

### Overweight/obesity enhanced fatty acid mobilization during sepsis

**Fig. 1:**
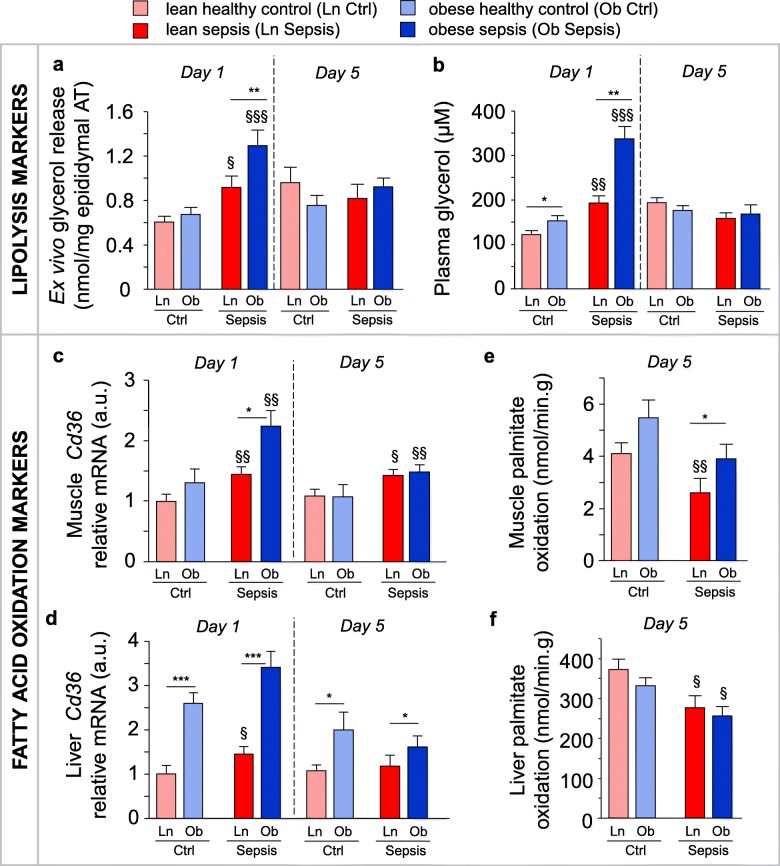
Effect of overweight/obesity on lipolysis and fatty acid oxidation during sepsis. Markers of fatty acid mobilization and metabolism were compared in lean (Ln) and overweight/obese (Ob) septic mice after 1 (d1) or 5 days (d5) of illness. **a** Ex vivo*-*released glycerol per epididymal adipose tissue (AT) explant mass. **b** Plasma glycerol. **c** Relative mRNA expression of fatty acid transporter *Cd36* in the muscle, and **d** the liver. **e** In vitro palmitate oxidation in the muscle and **f** the liver. Gene expression data are normalized to *Rn18s* or *Hprt* and shown relative to the mean of Ln healthy controls (Ctrl). **a**, **b**, **c**, **d** d1 Ctrl: Ln *n* = 15, Ob *n* = 10; d1 Sepsis: Ln *n =* 15, Ob *n =* 15; d5 Ctrl: Ln *n =* 17, Ob *n =* 15; d5 Sepsis: Ln *n =* 15, Ob *n* = 15. **e**, **f** Ctrl: Ln *n* = 18, Ob *n =* 15; Sepsis: Ln *n* = 14, Ob *n =* 15. Data are means ± SEM. *p* values determined through Wilcoxon or Student’s *t* test [Wilcoxon *p* values: **a** d1 *p* = 0.003, d5 *p* = 0.3, **c** d1 *p* = 0.008, d5 *p =* 0.003, **d** d1 *p* < 0.0001, d5 *p* = 0.05, **e** *p =* 0.003 ANOVA *p* values: **b** d1 *p* < 0.0001, d5 *p =* 0.3, **f** *p* = 0.007]. § *p* ≤ 0.05, §§ *p* ≤ 0.01, §§§ *p* ≤ 0.001 between Ctrl and Sepsis and * *p* ≤ 0.05, ** *p* ≤ 0.01, ***p ≤ 0.001 between sepsis groups

In the first study (Table 1), we hypothesized that overweight/obese mice have an altered metabolic response to sepsis compared to the lean mice, with elevated mobilization and metabolism of endogenous fatty acids. In overweight/obese mice, initial fat pad weight was higher than in the lean mice (910 ± 128 mg vs. 408 ± 36 mg; *p* = 0.01). During their illness, overweight/obese septic mice lost about twice the amount of fat than did lean septic mice (day 5 fat pad weight 462 ± 53 mg in the overweight/obese vs. 162 ± 42 mg in the lean; *p* = 0.0006). After 1 day of sepsis, plasma glycerol and ex vivo-released glycerol from adipose tissue (markers of lipolysis) were increased, which was more pronounced in overweight/obese than in lean mice (Fig. 1a,b). Nevertheless, during this acute phase of sepsis, both overweight/obese and lean septic mice presented with a similar plasma lipid profile as pair-fed controls, with low fatty acid, LDL- and HDL-cholesterol concentrations and normal triglyceride concentrations (Additional file 1: Figure S1). This suggested enhanced peripheral uptake of the released fatty acids during acute sepsis, especially in the overweight/obese mice. Indeed, sepsis-induced upregulation of fatty acid uptake transporter *Cd36* mRNA in muscle and liver was most pronounced in the overweight/obese (Fig. 1c,d). Expression of other markers of hepatic and muscular fatty acid oxidation was generally unaffected by obesity or sepsis (Additional file 1: Figure S1).

After 5 days of sepsis, plasma and ex vivo-released glycerol from adipose tissue returned to control levels in both overweight/obese and lean septic mice (Fig. 1a,b). Plasma fatty acid, triglyceride and LDL-cholesterol concentrations normalized, whereas plasma HDL-cholesterol concentrations remained low (Additional file 1: Figure S1). Nevertheless, hepatic and muscular *Cd36* gene expression were still higher with sepsis, for liver only in overweight/obese mice (Fig. 1c, d). Furthermore, in these overweight/obese prolonged septic mice, in vitro palmitate oxidation in the muscle, but not the liver, remained up to control levels. In contrast, in vitro palmitate oxidation was reduced in the lean (Fig. 1e,f). Also during prolonged sepsis, gene expression of markers of hepatic and muscle fatty acid oxidation were generally unaffected by obesity or sepsis (Additional file 1: Figure S1).

### Blocking fatty acid mobilization in overweight/obese mice caused sepsis-induced muscle wasting and weakness

**Fig. 2:**
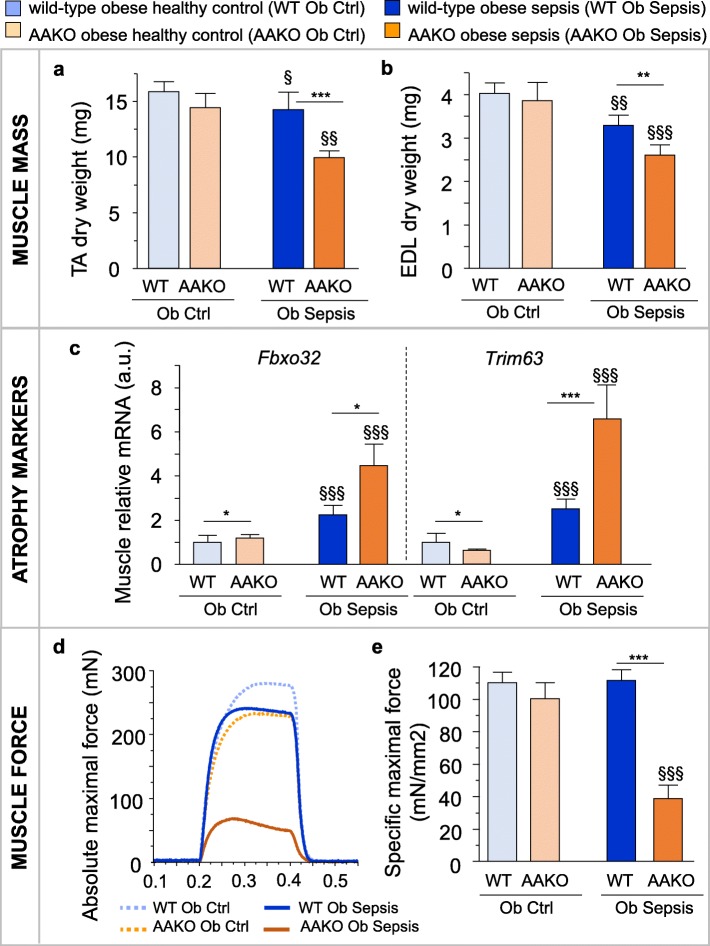
Blocking lipolysis aggravated muscle wasting and weakness in overweight/obese septic mice. Markers of muscle wasting and weakness were assessed in overweight/obese (Ob) septic wild-type (WT) and adipose tissue-specific ATL knockout (AAKO) mice after 5 days of illness. **a** Tibialis anterior (TA) and **b** extensor digitorum longus (EDL) muscle dry weight. **c** Relative mRNA expression of muscle atrophy markers. Gene expression data are normalized to *Rn18s* and presented relative to the mean of WT Ob healthy controls (Ctrl). **d** Representative force tracings of ex vivo*-*measured absolute maximal tetanic force of the EDL muscle. **e** Ex vivo force measurements of the EDL indicating the specific maximal force (peak tetanic tension per unit muscle mass). **a**, **b**, **c** Ob Ctrl: WT *n* = 19, AAKO *n =* 18; Ob Sepsis: WT *n =* 19, AAKO *n* = 17. **e** Ob Ctrl: WT *n =* 17, AAKO *n =* 14; Ob Sepsis: WT *n =* 14, AAKO *n* = 14. Data are means ± SEM. *p* values determined through Wilcoxon Test [Wilcoxon *p* values: **a** *p* < 0.0001, **b** *p* < 0.0001, **c** *Fbxo32 p* < 0.0001, *Trim63 p* < 0.0001, **e** *p* < 0.0001]. § *p* ≤ 0.05, §§ *p* ≤ 0.01, §§§ *p* ≤ 0.001 between Ctrl and Sepsis and * *p* ≤ 0.05, ** *p* ≤ 0.01, ***p ≤ 0.001 between sepsis groups

In the second study (Table 1), we hypothesized that overweight/obese septic mice would no longer be protected against the loss of muscle mass and function when they are unable to release fatty acids from their excess adipose tissue. Blocking lipolysis via AAKO in overweight/obese mice indeed caused a greater loss of TA and EDL muscle mass after 5 days of sepsis compared to wild-type overweight/obese mice (Fig. 2a,b). This more severe muscle wasting in the AAKO overweight/obese coincided with additional upregulation of the ubiquitin-proteasome system markers *Fbxo32* and *Trim63* (Fig. 2c). Furthermore, AAKO overweight/obese septic mice suffered from extremely reduced absolute (*p* < 0.0001 vs. AAKO healthy controls; Fig. 2d) and specific maximal muscle force (Fig. 2e). In contrast, wild-type overweight/obese septic mice displayed only slightly reduced absolute maximal force (*p* = 0.01 vs. wild-type healthy controls; Fig. 2d) and maintained specific maximal force up to healthy levels (Fig. 2e). AAKO overweight/obese septic mice also displayed a trend for a higher mortality rate than wild-type overweight/obese septic mice (Table 1) and had worse cumulative illness scores (10.1 ± 1.4 in AAKO vs. 5.4 ± 1.2 in wild-type; *p* = 0.02).

### Providing a lipid-rich parenteral infusion protected lean mice against sepsis-induced muscle weakness, but induced liver steatosis and a deranged lipid profile

**Fig. 3:**
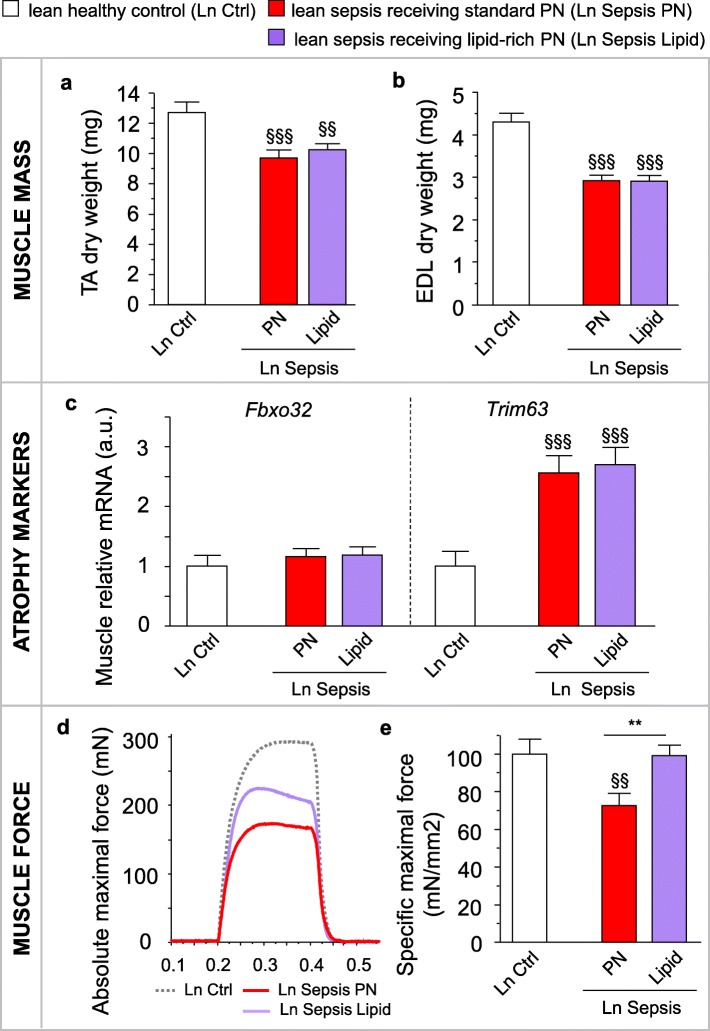
Providing mixed long- and medium-chain triglycerides to lean septic mice protected against muscle weakness. Lean (Ln) septic mice were given either standard mixed parenteral nutrition (PN) or a lipid-rich PN (Lipid) containing a mix of long- and medium-chain triglycerides for 5 days. **a** Tibialis anterior (TA) and **b** extensor digitorum longus (EDL) muscle dry weight. **c** Relative mRNA expression of muscle atrophy markers. Gene expression data are normalized to *Rn18s* and shown relative to the mean of Ln healthy control mice (Ctrl). **d** Representative force tracings of ex vivo*-*measured absolute maximal tetanic force of the EDL muscle. **e** Ex vivo force measurements of the EDL indicating the specific maximal force (peak tetanic tension per unit muscle mass). **a**, **b**, **c** Ln Ctrl *n* = 24; Ln Sepsis: PN *n* = 23, Lipid *n* = 23. **e** Ln Ctrl *n =* 17; Ln Sepsis: PN *n =* 16, Lipid *n* = 15. Data are means ± SEM. *p* values determined through Wilcoxon or Student’s *t* test [Wilcoxon *p* values: **c** *Fbxo32 p* = 0.2, *Trim63 p* < 0.0001; ANOVA *p* values: **a** *p* = 0.0008, **b** *p* < 0.0001, **e** *p* = 0.01]. § *p* ≤ 0.05, §§ *p* ≤ 0.01, §§§ *p* ≤ 0.001 between Ctrl and Sepsis and *p ≤ 0.05, **p ≤ 0.01, ***p ≤ 0.001 between Sepsis groups

Results from the first two studies in overweight/obese mice identified the increased availability of endogenously released lipids as key in the protection against sepsis-induced muscle wasting and weakness. In the third study (Table 1), we hypothesized that an increased lipid availability in lean septic mice would also protect them against sepsis-induced muscle wasting and weakness. However, after 5 days of sepsis, both lean mice that received standard mixed PN or lipid-rich PN presented with reduced TA and EDL muscle masses (Fig. 3a,b). Ubiquitin-proteasome system markers *Fbxo32* and *Trim63* mRNA were also comparably affected by sepsis in both groups of lean mice (Fig. 3c). As expected, lean septic mice on standard PN suffered from reduced absolute maximal muscle force (*p* < 0.001 vs. healthy controls; Fig. 3d) and specific maximal muscle force (Fig. 3e). In contrast, lean septic mice on lipid-rich PN showed less reduced absolute muscle force (*p* = 0.05 between standard PN and lipid-rich PN; Fig. 3d) and maintained their specific maximal muscle force equivalent to that of healthy controls (Fig. 3e).

**Fig. 4:**
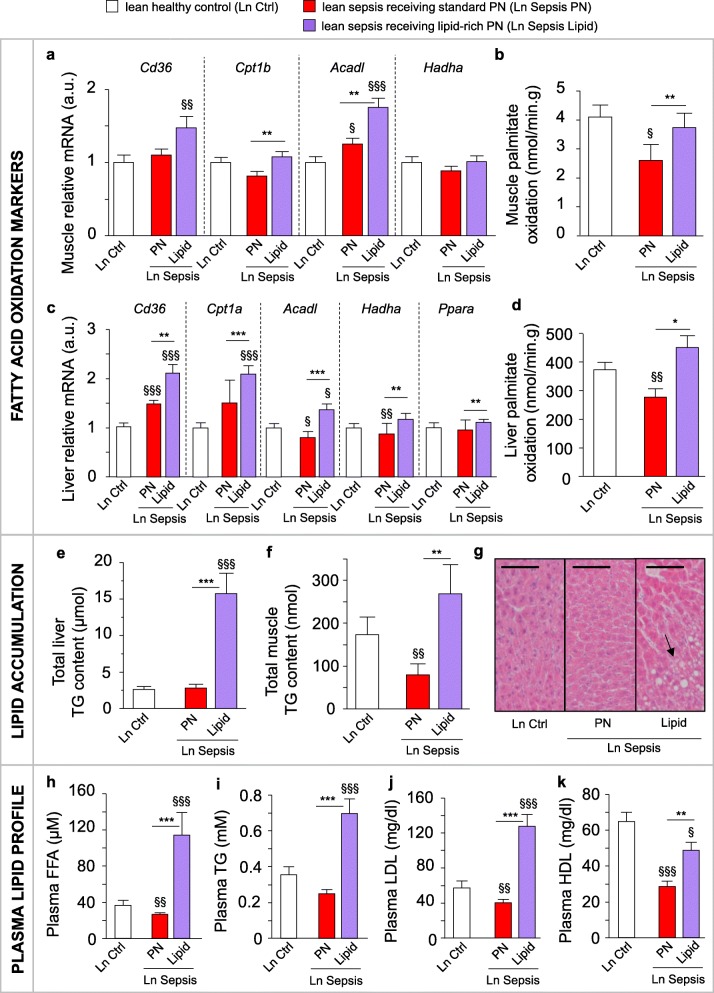
Providing mixed long- and medium-chain triglycerides to lean septic mice altered lipid metabolism. The effects on lipid metabolism of providing lean (Ln) septic with either standard mixed parenteral nutrition (PN) or a lipid-rich PN (Lipid) for 5 days. **a** Relative mRNA expression of genes involved in fatty acid oxidation in the muscle. **b** Muscle in vitro palmitate oxidation. **c** Relative mRNA expression of genes involved in hepatic fatty acid oxidation. **d** Hepatic in vitro palmitate oxidation. **e** Total hepatic triglyceride (TG) content (as μmol TG per total liver weight (mg)). **f** Total muscle TG content (as nmol TG per total muscle weight (mg)). **g** Liver histology, arrow indicates lipid accumulation. Scale bars are 50 μm. **h** Plasma free fatty acid (FFA), **i** TG, **j** LDL-cholesterol, and **k** HDL-cholesterol concentration. Gene expression data are normalized to *Rn18s* or *Hprt* and presented relative to the mean of Ln healthy control mice (Ctrl). **a**, **c**, **e**, **f**, **h**, **i**, **j**, **l** Ln Ctrl *n =* 24; Ln Sepsis: PN *n =* 23, Lipid *n =* 23. **b**, **d** Ln Ctrl *n =* 18; Ln Sepsis: PN *n =* 14, Lipid *n =* 14. Data are means ± SEM. *p* values determined through Wilcoxon or Student’s *t* test [Wilcoxon *p* values: **a** *Cd36 p* = 0.03, **b** *p =* 0.03, **c** *Ppara p* = 0.009, *Cpt1a p* < 0.0001, *Acadl p =* 0.0008, *Hadha p =* 0.01, **e** *p* < 0.0001, **f** *p =* 0.006, **h** *p* < 0.0001, **i** *p* < 0.0001, **j** *p* < 0.0001, **k** *p* < 0.0001; ANOVA *p* values: **a** *Cpt1b p =* 0.03, *Acadl p* < 0.0001, *Hadha p* = 0.4, **c** *Cd36 p* < 0.0001, **d** *p =* 0.003]. § *p* ≤ 0.05, §§ *p* ≤ 0.01, §§§ *p* ≤ 0.001 between Ctrl and Sepsis and * *p* ≤ 0.05, ** *p* ≤ 0.01, ***p ≤ 0.001 between Sepsis groups

Muscular and hepatic gene expression of fatty acid oxidation markers *Cd36*, *Cpt1b*, *Acadl*, and *Hadha*, and in vitro palmitate oxidation were highly increased in septic lean mice on lipid-rich PN compared to those on standard mixed PN (Fig. 4a–d). Importantly, giving high doses of lipids to lean septic mice did not adversely affect the mortality rate (Table 1), nor the cumulative illness scores (7.1 ± 1.1 with lipid-rich PN vs. 6.2 ± 0.9 with standard mixed PN; *p* = 0.7). However, the lipid-rich PN did cause triglyceride accumulation in both the liver and the muscle (Fig. 4e,f). Furthermore, these mice also presented with histologically confirmed liver steatosis (global steatosis score: 2.72 ± 1.54 with lipid-rich PN vs. 1.56 ± 1.03 with standard mixed PN; *p* = 0.01; Fig. 4g) and elevated plasma-free fatty acid, triglyceride, and LDL and HDL cholesterol concentrations (Fig. 4h–k).

### Enhanced lipid availability improved ketogenesis

**Fig. 5:**
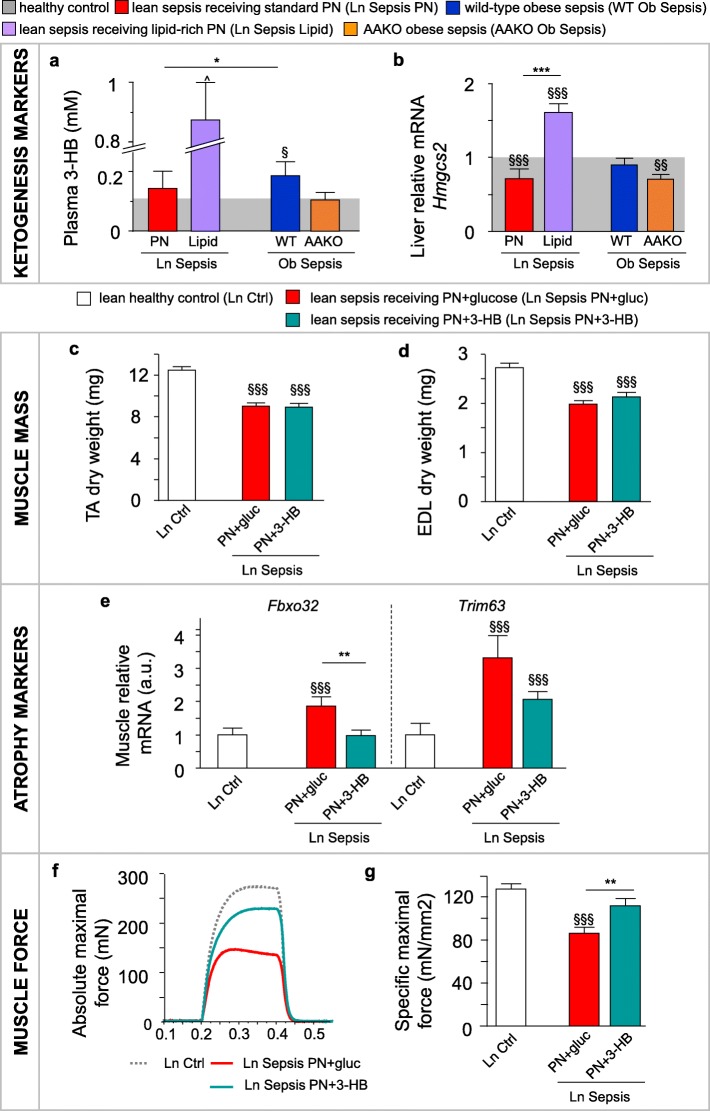
3-Hydroxybutyrate supplementation protects against sepsis-induced muscle weakness*.* The effect of an elevated lipid availability on ketogenesis was assessed after 5 days of sepsis. **a** Plasma 3-hydroxybutyrate (3-HB) concentration in lean (Ln) and obese (Ob) septic mice. **b** Hepatic gene expression of ketogenic enzyme *Hmgcs2*. Ln parenterally fed septic mice were supplemented with either 3-HB (PN+3-HB) or glucose (PN+gluc) for 5 days*.* **c** Tibialis anterior (TA) and **d** extensor digitorum longus (EDL) muscle dry weight. **e** Relative mRNA expression of muscle atrophy markers. **f** Representative force tracings of ex vivo*-*measured absolute maximal tetanic force of the EDL muscle. **g** Ex vivo-measured EDL specific maximal force (peak tetanic tension per unit muscle mass). mRNA data are normalized to *Rn18s* or *Hprt* and displayed relative to the mean of Ln Ctrl. **a** Light gray bar is the mean of all (Ln+Ob) healthy controls (Ctrl; *n* = 89); Sepsis: Ln *n* = 37, Ln Lipid *n =* 23, Ob *n* = 34, AAKO Ob *n =* 17. **b** Light gray bar is the mean of Ln (*n =* 24) and Ob (*n =* 18) Ctrl. Sepsis: Ln *n =* 23, Ln Lipid *n =* 23, Ob *n =* 19, AAKO Ob *n =* 17. **c**, **d**, **e** Ln Ctrl *n =* 15; Ln Sepsis: PN+gluc *n =* 17, PN+3-HB *n =* 17. **g** Ln Ctrl *n =* 15; Ln Sepsis: PN+gluc *n =* 16, PN+3-HB *n =* 14. Data are mean ± SEM. *p* values determined by Wilcoxon or Student’s *t* test [Wilcoxon *p* values: **a** *p* < 0.0001, **e** *Fbxo32 p* = 0.001, *Trim63 p* = 0.0002; ANOVA *p* values: **c** *p* < 0.0001, **d** *p* < 0.0001, **g** *p* < 0.0001]. § *p* ≤ 0.05, §§ *p* ≤ 0.01, §§§ *p* ≤ 0.001 between Ctrl and Sepsis and *p ≤ 0.05, **p ≤ 0.01, ***p ≤ 0.001 between Sepsis groups. ^*p* < 0.0001 compared to Ln Sepsis Lipid

In a previous study, the protection against sepsis-induced muscle wasting and weakness in overweight/obese mice coincided with elevated plasma concentration of ketone body 3-HB [15]. Here, we confirm the higher plasma 3-HB concentrations in overweight/obese septic mice than in the lean mice (Fig. 5a). Furthermore, these overweight/obese septic mice showed better preserved hepatic gene expression of the main ketogenic enzyme *Hmgcs2* (Fig. 5b). Blocking lipolysis via AAKO in overweight/obese septic mice prevented the rise in plasma 3-HB concentrations and lowered the hepatic *Hmgcs2* mRNA expression, similar as in lean septic mice (Fig. 5a,b). In contrast, providing lean septic mice with lipid-rich PN drastically increased hepatic *Hmgcs2* mRNA expression and plasma 3-HB concentrations (Fig. 5a,b). This elevated ketogenesis in the overweight/obese and in lean mice receiving lipid-rich PN was not due to a change in plasma insulin concentrations (Additional file 2: Figure S2). Combined, these results suggest that ketone bodies may play an important role in the protection against muscle wasting and weakness.

### 3-HB supplementation protected against sepsis-induced muscle weakness and enhanced muscle regeneration

In the fourth study (Table 1), we hypothesized that direct exogenous administration of ketone bodies can also protect lean septic mice against muscle wasting and weakness. After 5 days, all lean septic mice lost TA and EDL muscle mass, irrespective of 3-HB supplementation (Fig. 5c,d). However, 3-HB supplementation did prevent *Fbxo32* mRNA upregulation (Fig. 5e). Similar to the lipid-rich PN, 3-HB supplementation in lean septic mice attenuated muscle weakness, with less reduced absolute maximal muscle force (*p* = 0.002 for PN+3-HB vs. PN+gluc; Fig. 5f) and a specific maximal muscle force comparable to that of lean healthy control mice (Fig. 5g). PN+3-HB in lean septic mice did not affect mortality (Table 1) nor cumulative illness scores (8.1 ± 1.2 with PN+3-HB vs. 6.5 ± 1.3 with PN+gluc; *p* = 0.2). Unlike lipid-rich PN, PN+3-HB did not affect markers of fatty acid oxidation in muscle or liver (Additional file 3: Figure S3) or induce hepatic triglyceride accumulation (Additional file 3: Figure S3). Furthermore, 3-HB supplementation partially normalized the plasma lipid profile (Additional file 3: Figure S3). In the muscle of PN+3-HB septic mice, markers of ketolysis, the triglyceride and glycogen content, and plasma glucose concentrations were decreased or unaltered compared to PN+gluc (Additional file 3: Figure S3). Together, these results argue against the use of 3-HB as an alternative energy substrate by the muscle during critical illness.

**Fig. 6:**
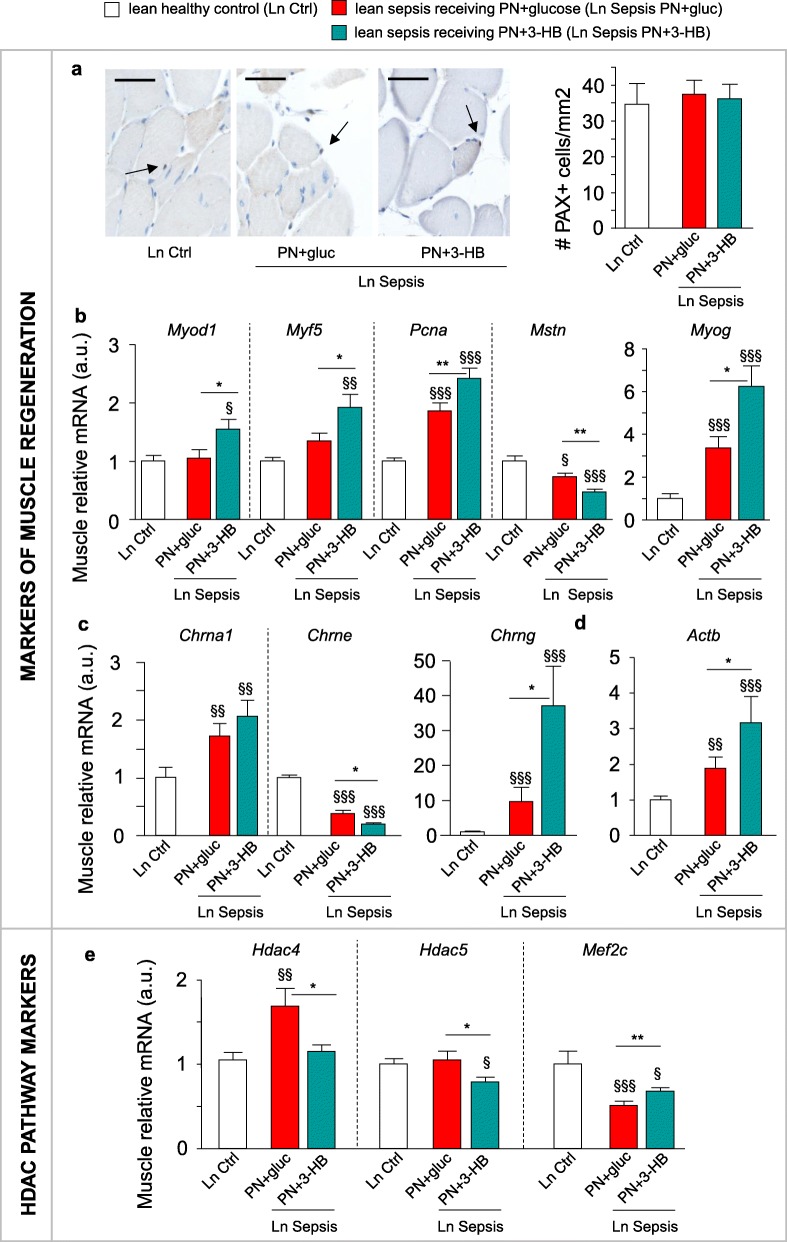
3-HB supplementation enhanced markers of muscle regeneration. Markers related to muscle regeneration were evaluated in lean (Ln) parenterally fed septic mice, supplemented with either 3-hydroxybutyrate (PN+3-HB) or glucose (PN+gluc) for 5 days. **a** Muscle PAX+ satellite cell staining and quantification of number of PAX+ cells per area. Arrow indicates PAX+ cells. Scale bar is 25 μm. **b** Relative muscular mRNA expression of genes involved in muscle regeneration, **c** genes encoding acetylcholine receptor subunits, **d** the beta-actin encoding gene, and **e** HDAC class II encoding genes and downstream targets. Gene expression data are normalized to *Rn18s* and presented relative to the mean of Ln healthy controls (Ctrl). All panels: Ln Ctrl *n =* 15; Ln Sepsis: PN+gluc *n =* 17, PN+3-HB *n =* 17. Data are means ± SEM. *p* values determined through Wilcoxon or Student’s *t* test [Wilcoxon *p* values: **a** *p* = 0.6, **b** *Myod1 p =* 0.03, *Myf5 p* = 0.01, *Mstn p* = 0.0002, *Myog p* < 0.0001, **c** *Chrna1 p* = 0.01, *Chrng p* < 0.0001, *Chrne p* < 0.0001, **d** *p* = 0.0002, **e** *Hdac4 p =* 0.01, *Mef2c p* = 0.0004; ANOVA *p* values: **b** *Pcna p* < 0.0001, **e** *Hdac5 p* = 0.05]. § *p* ≤ 0.05, §§ *p* ≤ 0.01, §§§ *p* ≤ 0.001 between Ctrl and Sepsis and * *p* ≤ 0.05, ** *p* ≤ 0.01, ***p ≤ 0.001 between Sepsis groups

Several signaling functions have been attributed to 3-HB as well: 3-HB has known anti-inflammatory and autophagy-activating properties and can stimulate mTOR-mediated protein synthesis. However, the illness-induced effects on these pathways were not altered by 3-HB supplementation (Additional file 4: Figure S4). In contrast, PN+3-HB did have effects on pathways involved in muscle regeneration. Whereas the number of PAX7-positive muscle satellite cells was unaffected by illness (Fig. 6a), gene expression of proliferation marker *Pcna* was clearly increased by sepsis, especially with PN+3-HB (Fig. 6b). As compared to PN+gluc, PN+3-HB increased the expression of regeneration markers *Myod1*, *Myog*, and *Myf5* in lean septic mice (Fig. 6b)*.* Furthermore, PN+3-HB caused a greater reduction in gene expression of myostatin, an inhibitor of muscle growth (Fig. 6b). Both groups of septic mice showed similarly increased expression of the muscle acetylcholine receptor (AchR) α-polypeptide gene (*Chrna1*) (Fig. 6c). This gene encodes for the α-subunit that is present in all AchRs. Expression of the adult AchR ε-subunit gene (*Chrne*) that is found in mature myofibers was most reduced in lean PN+3-HB septic mice (Fig. 6c). In contrast, PN+3-HB caused a four times higher increase than PN+gluc in the expression of the embryonic AchR γ-subunit gene (*Chrng*), found in immature myofibers (Fig. 6c). These 3-HB supplemented lean septic mice also presented with a greater elevation of *Actb* mRNA than the PN+gluc septic mice (Fig. 6d). Of note, histone deacetylases (HDAC) 4 and 5 are known inhibitors of the muscle regeneration pathway by blocking the expression of myogenic transcription factor MEF2. In lean septic mice receiving PN+3-HB, muscular expression of *Hdac4* and *Hdac5* was suppressed (Fig. 6e). Furthermore, *Mef2c* mRNA was higher in lean PN+3-HB than lean PN+gluc ill mice (Fig. 6e).

## Discussion

We here showed that overweight/obese septic mice had more pronounced lipolysis, peripheral fatty acid oxidation, and ketogenesis than did lean mice. Blocking lipolysis abolished the overweight/obesity-induced protection of the muscle, whereas high intravenous doses of lipids attenuated muscle weakness in lean septic mice. Although this nutritional strategy enhanced fatty acid oxidation and ketogenesis, it also caused adverse liver steatosis and a deranged lipid profile. Ketone body supplementation to PN in lean septic mice also attenuated muscle weakness and improved muscle regeneration without such adverse effects.

Critical illness in lean patients is characterized by impaired ketogenesis and reduced hepatic and muscular fatty acid metabolism [41–43]. Remarkably, overweight/obese septic mice did not display such impairment but maintained the typical metabolic profile present with diet-induced obesity (enhanced lipolysis and elevated hepatic fatty acid metabolism) [16–20, 22, 23]. Furthermore, this obesity-induced enhanced lipolysis appears crucial during sepsis. Indeed, blocking lipolysis in overweight/obese septic mice profoundly aggravated muscle wasting and weakness. Also in healthy non-obese mice, insufficient availability of adipose tissue-derived fatty acids via AAKO caused reduced exercise performance [44]. This demonstrates an important role of lipid availability for normal muscle function.

With these four consecutive animal studies, we have identified the mechanisms underlying the protection against sepsis-induced muscle wasting and weakness in overweight/obese mice. Whether a similar mechanism holds true for human patients requires further investigation. We previously demonstrated that after 1 week of critical illness, overweight/obese patients showed better preservation of myofiber size and suffered less from ICU-acquired weakness [15]. A small pilot feasibility study did confirm larger initial muscle depth in obese critically ill patients but failed to demonstrate a difference over time [45]. In general, the potential benefit of overweight/obesity in critically ill patients has mainly been investigated in relation to mortality. Better ICU survival has been observed in overweight/obese patients compared to those who are underweight, have a normal weight, or are morbidly obese [46–49]. As ICU-acquired weakness is an independent risk factor for death in the ICU [2, 50], the overweight/obesity-induced protection of the muscle may, to a certain extent, contribute to the observed survival benefit.

Both premorbid overweight/obesity and treatment with high lipid doses enhanced ketogenesis and resulted in elevated plasma 3-HB concentrations. Furthermore, 3-HB supplementation to PN in lean ill septic mice could mimic the protective effect of giving high lipid doses and of overweight/obesity on muscle weakness. This suggests that ketogenesis is key in protecting against sepsis-induced muscle weakness. In line with our findings, exogenous supplementation of ketone bodies has been shown to improve physical endurance performance in both rats and elite athletes [25, 51]. In contrast, other supplementation studies did not show enhanced physical performances in trained men [52–54].

Neither the supplementation of PN with 3-HB nor the infusion of high lipid doses in lean septic mice could replicate the observed overweight/obesity-induced protection against muscle wasting [15]. This suggests that the preservation of muscle mass in the overweight/obese is likely related to other pathways. Possibly, the adipokine leptin could be implicated. Indeed, leptin has been shown to increase muscle mass and to reduce muscle atrophy [55–57]. Leptin concentrations also strongly correlate with adiposity, resulting in higher concentrations in obesity [58]. Of note, in other neuromuscular disorders such as cancer cachexia, increasing plasma ketone body concentrations did have an effect on muscle mass [24, 59]. Furthermore, 3-HB infusion decreased whole-body phenylalanine-to-tyrosine degradation in LPS-induced inflammation in humans, but had no beneficial effect on protein metabolism in septic patients [60, 61]. Hence, the exact effect of 3-HB on muscle mass in critically ill patients should also be further investigated.

Nevertheless, the observation that PN+3-HB during sepsis directly reduced muscle weakness has clear clinical potential. Whereas a beneficial effect on muscle force was also observed with high lipid doses, the observed side effects limit the therapeutic potential of this type of nutrition. Furthermore, the skeletal muscle appears to be bio-energetically inert for delivered lipids during chronic critical illness [43]. This observation further argues for a direct effect of ketones on muscle weakness, rather than of increased lipid availability.

The muscle protection of PN+3-HB during sepsis does not appear to be related to its use as an energy substrate, but rather by its effects as a signaling molecule [28, 29, 62]. In contrast to cerebral or active muscle 3-HB uptake and oxidation, 3-HB uptake in the resting skeletal muscle of healthy individuals displays saturation kinetics [63]. This may explain why supplemented ketones appeared to function as signaling molecules rather than energy substrates in our sick mice. Indeed, 3-HB clearly increased markers of early muscle regeneration and decreased the expression of class IIa HDACs. These deacetylases are known suppressors of the regeneration pathway through suppression of MEF2 [64–67]. Further research is needed to evaluate a direct link between improved muscle regeneration and muscle function by 3-HB. It is also unclear whether 3-HB supplementation of PN in lean septic mice does not only prevent but also restore muscle function when weakness is present. Given that muscle regeneration is impaired in the human critically ill and hampers longer-term recovery [12], the observed effect of 3-HB on muscle regeneration may hold great promise also for optimizing longer-term recovery of patients.

An important limitation of our study is the use of a mouse model to study ICU-acquired weakness. Hence, translation to the human patient should be done with caution, given several species-specific differences [68]. Nevertheless, the model of sepsis-induced muscle wasting and weakness resembles human ICU-acquired weakness. The septic mice were also fluid-resuscitated and received antibiotics, analgesia, and nutritional support. However, they were not mechanically ventilated nor did they receive other routinely used pharmacological agents that may contribute to the development of ICU-acquired weakness in patients [4]. Second, for certain pathways, we could rely on gene expression data alone, which might not always be reflected in protein changes as well. Third, we did not assess whether specific macronutrients of the standard PN played either a synergistic or antagonistic role in the observed protection with 3-HB supplementation. Indeed, it was demonstrated earlier that early provision of PN worsened weakness and slowed down recovery [9, 69, 70], which was largely attributable to the administered proteins [71, 72].

## Conclusions

The obesity-induced muscle protection during sepsis was partly mediated by elevated mobilization and metabolism of endogenous fatty acids. Increased lipid availability in lean septic mice not only protected against muscle weakness, but also induced adverse side effects. Both overweight/obesity and a lipid-rich PN in lean mice stimulated ketogenesis. As direct supplementation of 3-HB to PN in lean septic mice also protected against muscle weakness, these ketones appear to play a key role in the observed muscle protection. Furthermore, PN+3-HB specifically activated muscle regeneration pathways, which may contribute to attenuated muscle weakness also in the longer term. Going from observations in overweight/obese septic mice to interventions in the lean mice, it is now clear that supplementation of 3-HB should be further investigated as a novel metabolic strategy to prevent debilitating muscle weakness in critically ill human patients.

## Additional files

Additional file 1: **Figure S1.** *Plasma lipid profile and markers of fatty acid oxidation in lean and overweight/obese mice.* The plasma lipid profile and gene expression markers of fatty acid oxidation were assessed in lean (Ln) and overweight/obese (Ob) mice after 1 (d1) or 5 days (d5) of sepsis. (a) Plasma free fatty acid (FFA), (b) triglyceride (TG), (c) LDL-cholesterol, and (d) HDL-cholesterol concentrations. (e) Relative mRNA expression of genes involved in fatty acid oxidation in the muscle. (f) Relative mRNA expression of genes involved in hepatic fatty acid oxidation. Gene expression data are normalized to *Rn18s* or *Hprt* and shown relative to the mean of Ln healthy controls (Ctrl). For all panels: d1 Ctrl: Ln *n* = 15, Ob *n* = 10; d1 Sepsis: Ln *n* = 15, Ob *n* = 15; d5 Ctrl: Ln *n* = 17, Ob *n* = 15; d5 Sepsis: Ln *n* = 15, Ob *n* = 15. Data are mean ± SEM. *p* values determined through Wilcoxon or Student’s *t* tests [Wilcoxon *p* values: (a) d1 *p* = 0.01, d5 *p* = 0.6, (b) d1 *p* = 0.5, d5 *p* = 0.5, (e) d1 *Acadl p* = 0.07, d1 *Hadha p* = 0.2, d5 *Hadha p* = 0.05, (f) d1 *Ppara p* = 0.7, d5 *Ppara p* < 0.0001, d1 *Cpt1a p* = 0.1, *d5 Cpt1a p* = 0.1, d5 *Acadl p* = 0.003, d5 *Hadha p* = 0.001; ANOVA *p* values: (c) d1 *p* < 0.0001, d5 *p* = 0.4, (d) d1 *p* < 0.0001, d5 *p* = 0.002, (e) d1 *Cpt1b p* = 0.1, d5 *Cpt1b p* = 0.1, d5 *Acadl p* = 0.002, (f) d1 *Acadl p* = 0.003, d1 *Hadha p* = 0.2]. § *p* ≤ 0.05, §§ *p* ≤ 0.01, §§§ *p* ≤ 0.001 between Ctrl and Sepsis, *p ≤ 0.05, **p ≤ 0.01, ***p ≤ 0.001 between sepsis groups. (DOCX 220 kb)

Additional file 2: **Figure S2.** *Plasma insulin*. Plasma insulin concentrations after 5 days of sepsis in (a) lean (Ln) and overweight/obese (Ob) mice and (b) in Ln mice receiving either standard mixed parenteral nutrition (PN), or a lipid-rich PN (Lipid). (a) Healthy control (Ctrl): Ln *n =* 17, Ob *n =* 15; d5 Sepsis: Ln *n =* 15, Ob *n =* 15. (b): Ln Ctrl *n =* 24; Ln Sepsis: PN *n =* 23, Lipid *n* = 23. Data are means ± SEM. *p* values determined through Wilcoxon or Student’s *t* test [Wilcoxon *p* values: (a) *p =* 0.1, (b) *p =* 0.01]. § *p* ≤ 0.05, §§ *p* ≤ 0.01, §§§ *p* ≤ 0.001 between Ctrl and Sepsis, * *p* ≤ 0.05, ** *p* ≤ 0.01, ***p ≤ 0.001 between sepsis groups. (DOCX 40 kb)

Additional file 3: **Figure S3.** *Ketone body 3-HB does not appear to function as alternative energy substrate during sepsis.* The effect of supplementation of glucose (PN+gluc) or 3-hydroxybutyrate (PN+3-HB) to lean (Ln) parenterally fed mice was evaluated after 5 days of sepsis. (a) Relative mRNA of genes involved in muscle and (b) hepatic fatty acid oxidation. (c) Total triglyceride (TG) content of the muscle (as nmol TG per total muscle weight (mg)) and (d) the liver (as μmol TG per total liver weight (mg)). (e) Plasma free fatty acid (FFA), (f) TG, (g) LDL-cholesterol, and (h) HDL-cholesterol concentration. (i) Relative mRNA of genes involved in ketolysis. (j) Plasma glucose concentrations. (k) Total glycogen content of the muscle (as μg glycogen per total muscle weight (mg)). Gene expression data are normalized to *Rn18s* or *Hprt* and presented relative to mean of Ln healthy controls (Ctrl). All panels: Ln Ctrl *n* = 15; Ln Sepsis: PN+gluc *n* = 17, PN+3-HB *n* = 17. Data are means ± SEM. *p* values determined through Wilcoxon or Student’s *t* test [Wilcoxon *p* values: (a) *Cd36 p =* 0.1, *Cpt1b p* = 0.6, *Acadl p* = 0.03, *Hadha p* = 0.3, (b) *Ppara p* = 0.002, *Cd36 p =* 0.05, (c) *p =* 0.001, (d) *p =* 0.6, (e) *p =* 0.2, (f) *p* = 0.004, (g) *p* = 0.01, (h) *p* < 0.0001, (i) *Mct1 p* = 0.0008, *Mct2 p* = 0.8, *Oxct1 p =* 0.01, (j) *p* < 0.0001 (k) *p =* 0.2; ANOVA *p* values: (b) *Cpt1a p =* 0.4, *Acadl p* < 0.0001, *Hadha p* < 0.0001]. § *p* ≤ 0.05, §§ *p* ≤ 0.01, §§§ *p* ≤ 0.001 between Ctrl and Sepsis, * *p* ≤ 0.05, ** *p* ≤ 0.01, ***p ≤ 0.001 between sepsis groups (DOCX 286 kb)

Additional file 4: **Figure S4.** *Effect of 3-HB on markers of autophagy, inflammation and mTOR-related protein synthesis.* The effect of supplementation of glucose (PN+gluc) or 3-hydroxybutyrate (PN+3-HB) to lean (Ln) parenterally fed mice was evaluated after 5 days of sepsis. (a) Relative expression of genes and (b) proteins involved in autophagy in the muscle. (c) Relative mRNA expression of genes involved in the inflammatory response in the muscle. (d) Plasma TNF-α concentration. (e) Relative expression of proteins involved in mTOR-related protein synthesis. Gene expression data are normalized to *Rn18s* or *Hprt* and presented relative to the mean of Ln healthy controls (Ctrl). Protein expression data are normalized to b-actin and displayed relative to the mean of Ln Ctrl. All panels: Ln Ctrl *n =* 15; Ln Sepsis: PN+gluc *n =* 17, PN+3-HB *n* = 17. Data are means ± SEM. *p* values determined through Wilcoxon Test [Wilcoxon *p* values: (a) *Atg5 p* = 0.01, *Atg7 p* < 0.0001, *Sqstm1 p* < 0.0001, (b) pULK1/ULK1 *p* = 0.7, p62 *p* = 0.0004, LC3 II/I *p =* 0.01, (c) *Tnfa p* = 0.005, *Il1b p* = 0.0006, *Nlrp3 p* < 0.0001, (d) *p* < 0.0001, (e) pS6K1/S6K1 *p* = 0.002, 4E-BP1γ/4E-BP1 *p =* 0.6]. § *p* ≤ 0.05, §§ *p* ≤ 0.01, §§§ *p* ≤ 0.001 between Ctrl and Sepsis, * *p* ≤ 0.05, ** *p* ≤ 0.01, ***p ≤ 0.001 between sepsis groups (DOCX 101 kb)

Additional file 5: **Figure S5.** *Confirmation of adipose tissue-specific ATGL knockout.* Markers of adipose tissue-specific ATGL knockout (AAKO) were assessed in overweight/obese (Ob) wild-type (WT) and knockout mice. (a) Relative mRNA expression of *Pnpla2/Atgl* in visceral (visc.) and subcutaneous (s.c.) adipose tissue (AT), muscle and liver. (b) Summation of visc., s.c., and epididymal AT depot weights after 5 days, as percentage of initial body weight. (c) Plasma glycerol concentration. (d) Ex vivo glycerol release per epididymal AT explant mass. (e) Plasma free fatty acid (FFA) concentration. (f) Relative mRNA expression of genes involved in hepatic fatty acid oxidation. Gene expression data are normalized to *Rn18s* or *Hprt* and presented relative to the mean of WT Ob healthy controls (Ctrl). For all panels: Ob Ctrl: WT *n =* 19, AAKO *n* = 18; Ob Sepsis: WT *n =* 19, AAKO *n =* 17. Data are mean ± SEM. *p* values determined through Wilcoxon or Student’s *t* test [Wilcoxon *p* values: (a) visc. AT *p* < 0.0001, s.c. AT *p* < 0.0001, muscle *p* < 0.0001, liver *p =* 0.002, (d) *p* < 0.0001, (f) *Ppara p* = 0.0002, *Cd36 p* < 0.0001, *Cpt1a p =* 0.01; ANOVA *p* values: (b) *p* < 0.0001, (c) *p* < 0.0001, (e) *p* = 0.003, (f) *Acadl p* = 0.9, *Hadha p* = 0.07]. § *p* ≤ 0.05, §§ *p* ≤ 0.01, §§§ *p* ≤ 0.001 between Ctrl and Sepsis, * *p* ≤ 0.05, ** *p* ≤ 0.01, ***p ≤ 0.001 between sepsis groups. (DOCX 219 kb)

Additional file 6: **Table S1.** List of commercial TaqMan® assays used for gene analyses. (DOCX 17 kb)

Additional file 7: Supplemental Material and Methods. (DOCX 83 kb)

## Data Availability

The datasets used and/or analyzed during the current study are available from the corresponding author on reasonable request.

## Abbreviations

3-HB: 3-Hydroxybutyrate
AAKO: Adipose tissue-specific ATGL knockout
AchR: Acetylcholine receptor
ATGL: Adipose triglyceride lipase
EDL: Extensor digitorum longus
HDAC: Histone deacetylase
ICU: Intensive care unit
PN: Parenteral nutrition
TA: Tibialis anterior
