# Supplementary material for: Adipose tissue protects against sepsis-induced muscle weakness in mice: from lipolysis to ketones
Source: Crit Care. 2019 Jul 1;23:236. doi: 10.1186/s13054-019-2506-6 (PMC6600878; doi:10.1186/s13054-019-2506-6)
Supplement: Supplementary file 1 — Figure S1. Plasma lipid profile and markers of fatty acid oxidation in lean and overweight/obese mice. The plasma lipid profile and gene expression markers of fatty acid oxidation were assessed in lean (Ln) and overweight/obese (Ob) mice after 1 (d1) or 5 days (d5) of sepsis. (a) Plasma free fatty acid (FFA), (b) triglyceride (TG), (c) LDL-cholesterol, and (d) HDL-cholesterol concentrations. (e) Relative mRNA expression of genes involved in fatty acid oxidation in the muscle. (f) Relative mRNA expression of genes involved in hepatic fatty acid oxidation. Gene expression data are normalized to Rn18s or Hprt and shown relative to the mean of Ln healthy controls (Ctrl). For all panels: d1 Ctrl: Ln n = 15, Ob n = 10; d1 Sepsis: Ln n = 15, Ob n = 15; d5 Ctrl: Ln n = 17, Ob n = 15; d5 Sepsis: Ln n = 15, Ob n = 15. Data are mean ± SEM. p values determined through Wilcoxon or Student’s t tests [Wilcoxon p values: (a) d1 p = 0.01, d5 p = 0.6, (b) d1 p = 0.5, d5 p = 0.5, (e) d1 Acadl p = 0.07, d1 Hadha p = 0.2, d5 Hadha p = 0.05, (f) d1 Ppara p = 0.7, d5 Ppara p < 0.0001, d1 Cpt1a p = 0.1, d5 Cpt1a p = 0.1, d5 Acadl p = 0.003, d5 Hadha p = 0.001; ANOVA p values: (c) d1 p < 0.0001, d5 p = 0.4, (d) d1 p < 0.0001, d5 p = 0.002, (e) d1 Cpt1b p = 0.1, d5 Cpt1b p = 0.1, d5 Acadl p = 0.002, (f) d1 Acadl p = 0.003, d1 Hadha p = 0.2]. § p ≤ 0.05, §§ p ≤ 0.01, §§§ p ≤ 0.001 between Ctrl and Sepsis, *p ≤ 0.05, **p ≤ 0.01, ***p ≤ 0.001 between sepsis groups. (DOCX 220 kb) [file 13054_2019_2506_MOESM1_ESM.docx]

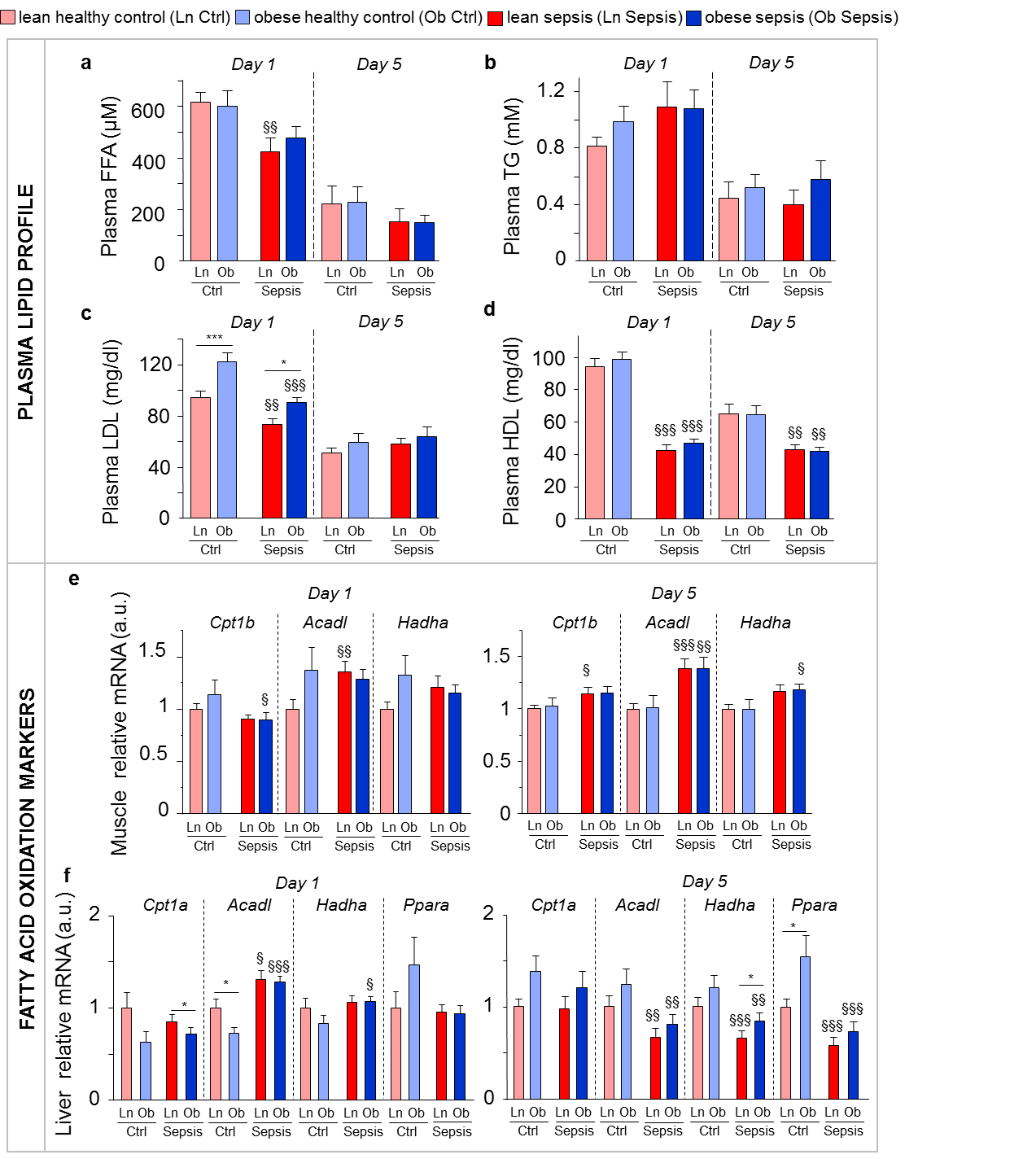


**Figure S1** *Plasma lipid profile and markers of fatty acid oxidation in lean and overweight/obese mice.* The plasma lipid profile and gene expression markers of fatty acid oxidation were assessed in lean (Ln) and overweight/obese (Ob) mice after 1 (d1) or 5 days (d5) of sepsis. (**a**) Plasma free fatty acid (FFA), (**b**) triglyceride (TG), (**c**) LDL-cholesterol, and (**d**) HDL-cholesterol concentrations. (**e**) Relative mRNA expression of genes involved in fatty acid oxidation in the muscle and (**f**) the liver. Gene expression data are normalized to *Rn18s* or *Hprt* and shown relative to the mean of Ln healthy controls (Ctrl). For all panels: d1 Ctrl: Ln n=15, Ob n=10; d1 Sepsis: Ln n=15, Ob n=15; d5 Ctrl: Ln n=17, Ob n=15; d5 Sepsis: Ln n=15, Ob n=15. Data are mean ± SEM. P-values determined through Wilcoxon or Student’s t Tests [Wilcoxon p-values: (**a**) d1 p=0.01, d5 p=0.6, (**b**) d1 p=0.5, d5 p=0.5, (**e**) d1 *Acadl* p=0.07, d1 *Hadha* p=0.2, d5 *Hadha* p=0.05, (**f**) d1 *Ppara* p=0.7, d5 *Ppara* p<0.0001, d1 *Cpt1a* p=0.1, *d5* *Cpt1a* p=0.1, d5 *Acadl* p=0.003, d5 *Hadha* p=0.001; ANOVA p-values: (**c**) d1 p<0.0001, d5 p=0.4, (**d**) d1 p<0.0001, d5 p=0.002, (**e**) d1 *Cpt1b* p=0.1, d5 *Cpt1b* p=0.1, d5 *Acadl* p=0.002, (**f**) d1 *Acadl* p=0.003, d1 *Hadha* p=0.2]. § p≤0.05, §§ p≤0.01, §§§ p≤0.001 between Ctrl and Sepsis, * p≤0.05, ** p≤0.01, ***p≤0.001 between Sepsis groups
