# Supplementary material for: Adipose tissue protects against sepsis-induced muscle weakness in mice: from lipolysis to ketones
Source: Crit Care. 2019 Jul 1;23:236. doi: 10.1186/s13054-019-2506-6 (PMC6600878; doi:10.1186/s13054-019-2506-6)

**Figure S2** *Plasma insulin.* Plasma insulin concentrations after 5 days of sepsis in (**a**) lean (Ln) and overweight/obese (Ob) mice and (**b**) in Ln mice receiving either standard mixed parenteral nutrition (PN), or a lipid-rich PN (Lipid). (**a**) Healthy control (Ctrl): Ln n=17, Ob n=15; d5 Sepsis: Ln n=15, Ob n=15. (**b**): Ln Ctrl n=24; Ln Sepsis: PN n=23, Lipid n=23. Data are means ± SEM. P-values determined through Wilcoxon or Student’s t Test [Wilcoxon p-values: (**a**) p=0.1, (**b**) p=0.01]. § p≤0.05, §§ p≤0.01, §§§ p≤0.001 between Ctrl and Sepsis, * p≤0.05, ** p≤0.01, ***p≤0.001 between Sepsis groups


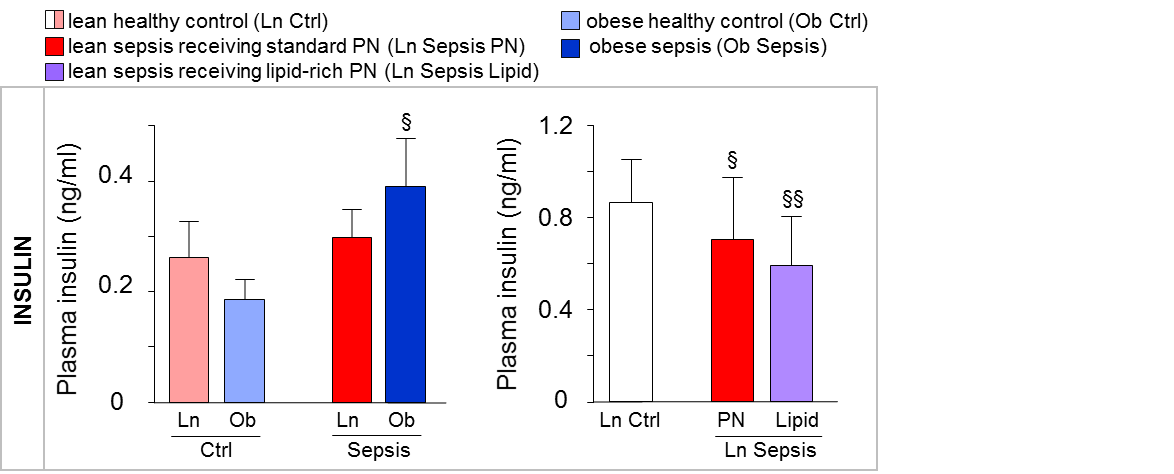

Supplement: Supplementary file 2 — Figure S2. Plasma insulin. Plasma insulin concentrations after 5 days of sepsis in (a) lean (Ln) and overweight/obese (Ob) mice and (b) in Ln mice receiving either standard mixed parenteral nutrition (PN), or a lipid-rich PN (Lipid). (a) Healthy control (Ctrl): Ln n = 17, Ob n = 15; d5 Sepsis: Ln n = 15, Ob n = 15. (b): Ln Ctrl n = 24; Ln Sepsis: PN n = 23, Lipid n = 23. Data are means ± SEM. p values determined through Wilcoxon or Student’s t test [Wilcoxon p values: (a) p = 0.1, (b) p = 0.01]. § p ≤ 0.05, §§ p ≤ 0.01, §§§ p ≤ 0.001 between Ctrl and Sepsis, * p ≤ 0.05, ** p ≤ 0.01, ***p ≤ 0.001 between sepsis groups. (DOCX 40 kb) [file 13054_2019_2506_MOESM2_ESM.docx]
