# Supplementary material for: Adipose tissue protects against sepsis-induced muscle weakness in mice: from lipolysis to ketones
Source: Crit Care. 2019 Jul 1;23:236. doi: 10.1186/s13054-019-2506-6 (PMC6600878; doi:10.1186/s13054-019-2506-6)

**Figure S3** *Ketone body 3-HB does not appear to function as alternative energy substrate during sepsis.* The effect of supplementation of glucose (PN+gluc) or 3-hydroxybutyrate (PN+3-HB) to lean (Ln) parenterally fed mice was evaluated after 5 days of sepsis. (**a**) Relative mRNA of genes involved in muscle and (**b**) hepatic fatty acid oxidation. (**c**) Total triglyceride (TG) content of the muscle (as nmol TG per total muscle weight (mg)) and (**d**) the liver (as µmol TG per total liver weight (mg)). (**e**) Plasma free fatty acid (FFA), (**f**) TG, (**g**) LDL-cholesterol, and (**h**) HDL-cholesterol concentration. (**i**) Relative mRNA of genes involved in ketolysis. (**j**) Plasma glucose concentrations. (**k**) Total glycogen content of the muscle (as µg glycogen per total muscle weight (mg)). Gene expression data are normalized to *Rn18s* or *Hprt* and presented relative to mean of Ln healthy controls (Ctrl). All panels: Ln Ctrl n=15; Ln Sepsis: PN+gluc n=17, PN+3-HB n=17. Data are means ± SEM. P-values determined through Wilcoxon or Student’s t Test [Wilcoxon p-values: (**a**) *Cd36* p=0.1, *Cpt1b* p=0.6, *Acadl* p=0.03, *Hadha* p=0.3, (**b**) *Ppara* p=0.002, *Cd36* p=0.05, (**c**) p=0.001, (**d**) p=0.6, (**e**) p=0.2, (**f**) p=0.004, (**g**) p=0.01, (**h**) p<0.0001, (**i**) *Mct1* p=0.0008, *Mct2* p=0.8, *Oxct1* p=0.01, (**j**) p<0.0001 (**k**) p=0.2; ANOVA p-values: (**b**) *Cpt1a* p=0.4, *Acadl* p<0.0001, *Hadha* p<0.0001]. § p≤0.05, §§ p≤0.01, §§§ p≤0.001 between Ctrl and Sepsis, * p≤0.05, ** p≤0.01, ***p≤0.001 between Sepsis groups


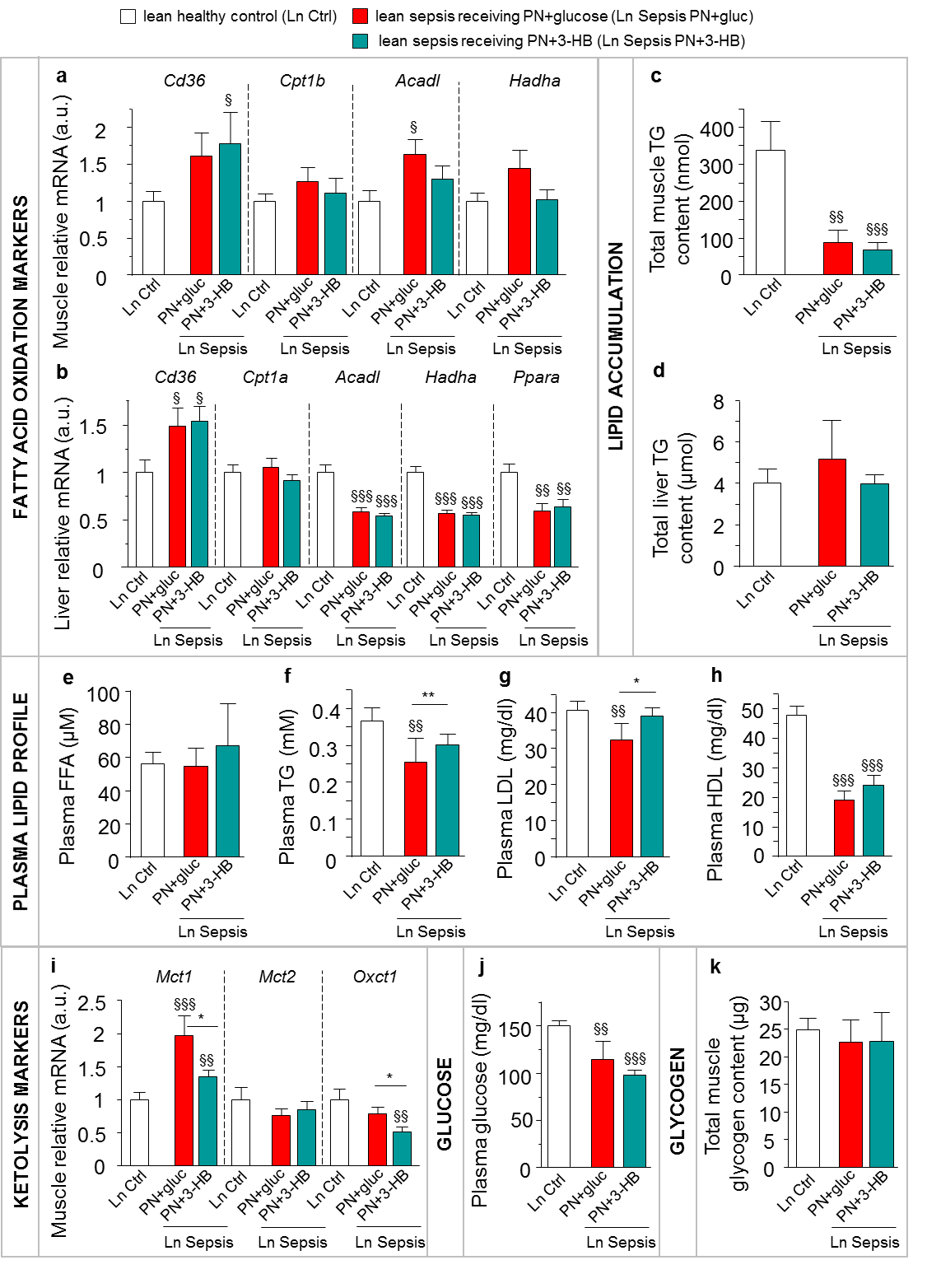

Supplement: Supplementary file 3 — Figure S3. Ketone body 3-HB does not appear to function as alternative energy substrate during sepsis. The effect of supplementation of glucose (PN+gluc) or 3-hydroxybutyrate (PN+3-HB) to lean (Ln) parenterally fed mice was evaluated after 5 days of sepsis. (a) Relative mRNA of genes involved in muscle and (b) hepatic fatty acid oxidation. (c) Total triglyceride (TG) content of the muscle (as nmol TG per total muscle weight (mg)) and (d) the liver (as μmol TG per total liver weight (mg)). (e) Plasma free fatty acid (FFA), (f) TG, (g) LDL-cholesterol, and (h) HDL-cholesterol concentration. (i) Relative mRNA of genes involved in ketolysis. (j) Plasma glucose concentrations. (k) Total glycogen content of the muscle (as μg glycogen per total muscle weight (mg)). Gene expression data are normalized to Rn18s or Hprt and presented relative to mean of Ln healthy controls (Ctrl). All panels: Ln Ctrl n = 15; Ln Sepsis: PN+gluc n = 17, PN+3-HB n = 17. Data are means ± SEM. p values determined through Wilcoxon or Student’s t test [Wilcoxon p values: (a) Cd36 p = 0.1, Cpt1b p = 0.6, Acadl p = 0.03, Hadha p = 0.3, (b) Ppara p = 0.002, Cd36 p = 0.05, (c) p = 0.001, (d) p = 0.6, (e) p = 0.2, (f) p = 0.004, (g) p = 0.01, (h) p < 0.0001, (i) Mct1 p = 0.0008, Mct2 p = 0.8, Oxct1 p = 0.01, (j) p < 0.0001 (k) p = 0.2; ANOVA p values: (b) Cpt1a p = 0.4, Acadl p < 0.0001, Hadha p < 0.0001]. § p ≤ 0.05, §§ p ≤ 0.01, §§§ p ≤ 0.001 between Ctrl and Sepsis, * p ≤ 0.05, ** p ≤ 0.01, ***p ≤ 0.001 between sepsis groups (DOCX 286 kb) [file 13054_2019_2506_MOESM3_ESM.docx]
