# Supplementary material for: Adipose tissue protects against sepsis-induced muscle weakness in mice: from lipolysis to ketones
Source: Crit Care. 2019 Jul 1;23:236. doi: 10.1186/s13054-019-2506-6 (PMC6600878; doi:10.1186/s13054-019-2506-6)
Supplement: Supplementary file 4 — Figure S4. Effect of 3-HB on markers of autophagy, inflammation and mTOR-related protein synthesis. The effect of supplementation of glucose (PN+gluc) or 3-hydroxybutyrate (PN+3-HB) to lean (Ln) parenterally fed mice was evaluated after 5 days of sepsis. (a) Relative expression of genes and (b) proteins involved in autophagy in the muscle. (c) Relative mRNA expression of genes involved in the inflammatory response in the muscle. (d) Plasma TNF-α concentration. (e) Relative expression of proteins involved in mTOR-related protein synthesis. Gene expression data are normalized to Rn18s or Hprt and presented relative to the mean of Ln healthy controls (Ctrl). Protein expression data are normalized to b-actin and displayed relative to the mean of Ln Ctrl. All panels: Ln Ctrl n = 15; Ln Sepsis: PN+gluc n = 17, PN+3-HB n = 17. Data are means ± SEM. p values determined through Wilcoxon Test [Wilcoxon p values: (a) Atg5 p = 0.01, Atg7 p < 0.0001, Sqstm1 p < 0.0001, (b) pULK1/ULK1 p = 0.7, p62 p = 0.0004, LC3 II/I p = 0.01, (c) Tnfa p = 0.005, Il1b p = 0.0006, Nlrp3 p < 0.0001, (d) p < 0.0001, (e) pS6K1/S6K1 p = 0.002, 4E-BP1γ/4E-BP1 p = 0.6]. § p ≤ 0.05, §§ p ≤ 0.01, §§§ p ≤ 0.001 between Ctrl and Sepsis, * p ≤ 0.05, ** p ≤ 0.01, ***p ≤ 0.001 between sepsis groups (DOCX 101 kb) [file 13054_2019_2506_MOESM4_ESM.docx]

**
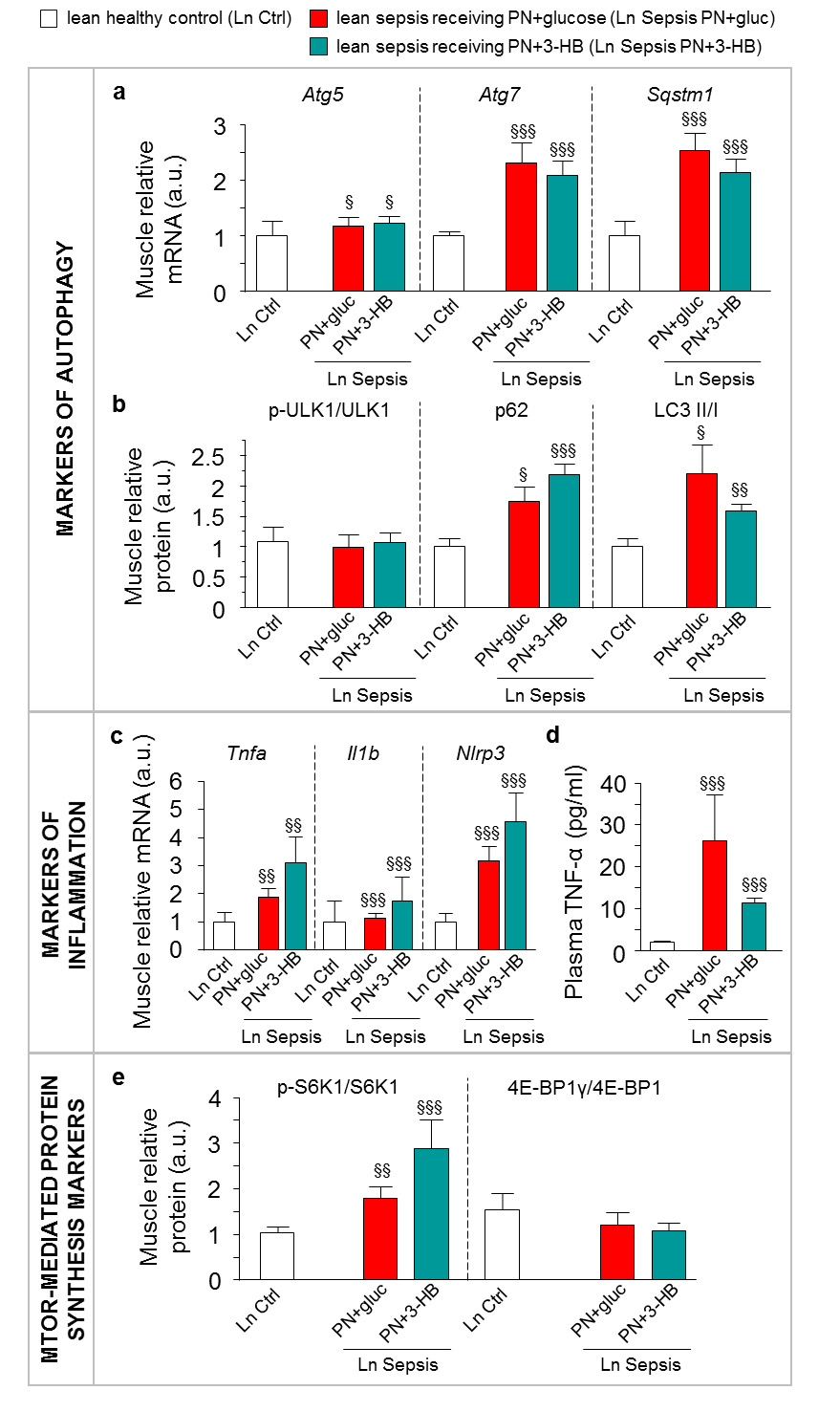
**

**Figure S4** *Effect of 3-HB on markers of autophagy, inflammation and mTOR-related protein synthesis.* The effect of supplementation of glucose (PN+gluc) or 3-hydroxybutyrate (PN+3-HB) to lean (Ln) parenterally fed mice was evaluated after 5 days of sepsis. (**a**) Relative expression of genes and (**b**) proteins involved in autophagy in the muscle. (**c**) Relative mRNA expression of genes involved in the inflammatory response in the muscle. (**d**) Plasma TNF-α concentration. (**e**) Relative expression of proteins involved in mTOR-related protein synthesis. Gene expression data are normalized to *Rn18s* or *Hprt* and presented relative to the mean of Ln healthy controls (Ctrl). Protein expression data are normalized to b-actin and displayed relative to the mean of Ln Ctrl. All panels: Ln Ctrl n=15; Ln Sepsis: PN+gluc n=17, PN+3-HB n=17. Data are means ± SEM. P-values determined through Wilcoxon Test [Wilcoxon p-values: (**a**) *Atg5* p=0.01, *Atg7* p<0.0001, *Sqstm1* p<0.0001, (**b**) pULK1/ULK1 p=0.7, p62 p=0.0004, LC3 II/I p=0.01, (**c**) *Tnfa* p=0.005, *Il1b* p=0.0006, *Nlrp3* p<0.0001, (**d**) p<0.0001, (**e**) pS6K1/S6K1 p=0.002, 4E-BP1γ/4E-BP1 p=0.6]. § p≤0.05, §§ p≤0.01, §§§ p≤0.001 between Ctrl and Sepsis, * p≤0.05, ** p≤0.01, ***p≤0.001 between Sepsis groups
