# Supplementary material for: Adipose tissue protects against sepsis-induced muscle weakness in mice: from lipolysis to ketones
Source: Crit Care. 2019 Jul 1;23:236. doi: 10.1186/s13054-019-2506-6 (PMC6600878; doi:10.1186/s13054-019-2506-6)
Supplement: Supplementary file 5 — Figure S5. Confirmation of adipose tissue-specific ATGL knockout. Markers of adipose tissue-specific ATGL knockout (AAKO) were assessed in overweight/obese (Ob) wild-type (WT) and knockout mice. (a) Relative mRNA expression of Pnpla2/Atgl in visceral (visc.) and subcutaneous (s.c.) adipose tissue (AT), muscle and liver. (b) Summation of visc., s.c., and epididymal AT depot weights after 5 days, as percentage of initial body weight. (c) Plasma glycerol concentration. (d) Ex vivo glycerol release per epididymal AT explant mass. (e) Plasma free fatty acid (FFA) concentration. (f) Relative mRNA expression of genes involved in hepatic fatty acid oxidation. Gene expression data are normalized to Rn18s or Hprt and presented relative to the mean of WT Ob healthy controls (Ctrl). For all panels: Ob Ctrl: WT n = 19, AAKO n = 18; Ob Sepsis: WT n = 19, AAKO n = 17. Data are mean ± SEM. p values determined through Wilcoxon or Student’s t test [Wilcoxon p values: (a) visc. AT p < 0.0001, s.c. AT p < 0.0001, muscle p < 0.0001, liver p = 0.002, (d) p < 0.0001, (f) Ppara p = 0.0002, Cd36 p < 0.0001, Cpt1a p = 0.01; ANOVA p values: (b) p < 0.0001, (c) p < 0.0001, (e) p = 0.003, (f) Acadl p = 0.9, Hadha p = 0.07]. § p ≤ 0.05, §§ p ≤ 0.01, §§§ p ≤ 0.001 between Ctrl and Sepsis, * p ≤ 0.05, ** p ≤ 0.01, ***p ≤ 0.001 between sepsis groups. (DOCX 219 kb) [file 13054_2019_2506_MOESM5_ESM.docx]

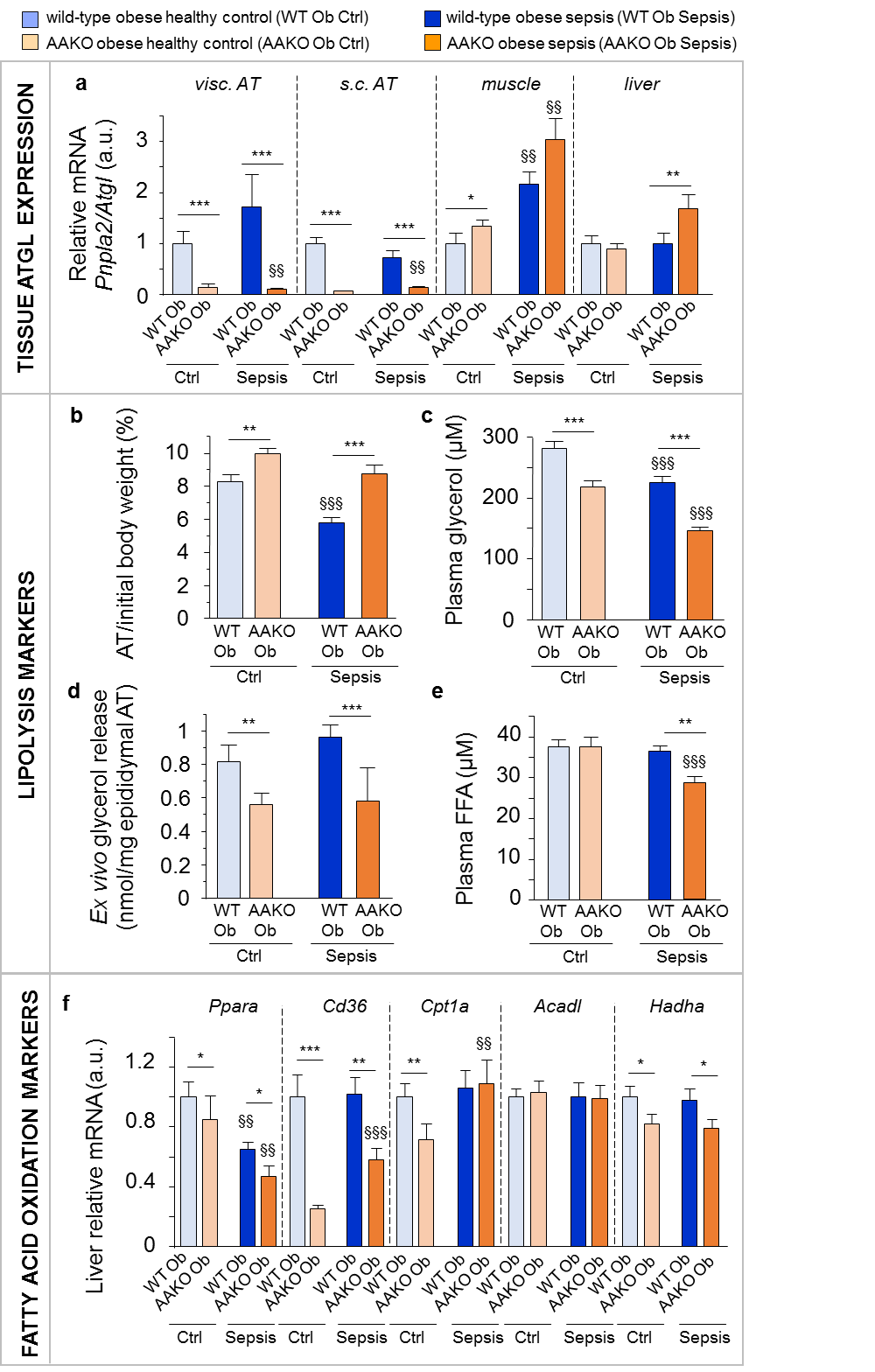


**Figure S5** *Confirmation of adipose tissue-specific ATGL knockout.* Markers of adipose tissue specific ATGL knockout (AAKO) were assessed in overweight/obese (Ob) wild-type (WT) and knockout mice. (**a**) Relative mRNA expression of *Pnpla2/Atgl* in visceral (visc.) and subcutaneous (s.c.) adipose tissue (AT), muscle and liver. (**b**) Summation of visc., s.c., and epididymal AT depot weights after 5 days, as percentage of initial body weight. (**c**) Plasma glycerol concentration. (**d**) E*x vivo* glycerol release per epididymal AT explant mass. (**e**) Plasma free fatty acid (FFA) concentration. (**f**) Relative mRNA expression of genes involved in hepatic fatty acid oxidation. Gene expression data are normalized to *Rn18s* or *Hprt* and presented relative to the mean of WT Ob healthy controls (Ctrl). For all panels: Ob Ctrl: WT n=19, AAKO n=18; Ob Sepsis: WT n=19, AAKO n=17. Data are mean ± SEM. P-values determined through Wilcoxon or Student’s t Test [Wilcoxon p-values: (**a**) visc. AT p<0.0001, s.c. AT p<0.0001, muscle p<0.0001, liver p=0.002, (**d**) p<0.0001, (**f**) *Ppara* p=0.0002, *Cd36* p<0.0001, *Cpt1a* p=0.01; ANOVA p-values: (**b**) p<0.0001, (**c**) p<0.0001, (**e**) p=0.003, (**f**) *Acadl* p=0.9, *Hadha* p=0.07]. § p≤0.05, §§ p≤0.01, §§§ p≤0.001 between Ctrl and Sepsis, * p≤0.05, ** p≤0.01, ***p≤0.001 between Sepsis groups
