# Supplementary material for: Adipose tissue protects against sepsis-induced muscle weakness in mice: from lipolysis to ketones
Source: Crit Care. 2019 Jul 1;23:236. doi: 10.1186/s13054-019-2506-6 (PMC6600878; doi:10.1186/s13054-019-2506-6)
Supplement: Supplementary file 6 — Table S1. List of commercial TaqMan® assays used for gene analyses. (DOCX 17 kb) [file 13054_2019_2506_MOESM6_ESM.docx]

**Table S1**: List of commercial TaqMan® assays used for gene analyses.

| ***Gene*** | ***Assay ID*** |
| --- | --- |
| ribosomal 18S (*Rn18s*) | Mm04277571_s1 |
| hypoxanthine guanine phosphoribosyl transferase (*Hprt*) | Mm03024075_m1 |
| peroxisome proliferator-activated receptor alpha (*Ppara*) | Mm00440939_m1 |
| cluster of differentiation 36 (*Cd36*) | Mm00432403_m1 |
| carnitine palmitoyltransferase I alpha (*Cpt1a*) | Mm01231183_m1 |
| carnitine palmitoyltransferase I beta (*Cpt1b)* | Mm00487191_g1 |
| long-chain acyl-CoA dehydrogenase (*Acadl*) | Mm00599660_m1 |
| hydroxyacyl-CoA dehydrogenase/3-ketoacyl-CoA thiolase/enoyl-CoA hydratase (*Hadha*) | Mm00805228_m1 |
| 3-hydroxy-3-methylglutaryl-Coenzyme A synthase 2 (*Hmgcs2*) | Mm00550050_m1 |
| patatin-like phospholipase domain containing 2 (*Pnpla2*) | Mm00503040_m1 |
| F-box protein 32 (*Fbxo32*) | Mm00499523_m1 |
| tripartite motif-containing 63 (*Trim63*) | Mm01185221_m1 |
| monocarboxylate transporter 1 (*Mct1*) | Mm01306379_m1 |
| monocarboxylate transporter 2 (*Mct2*) | Mm00441442_m1 |
| 3-oxoacid CoA-transferase 1 (*Oxct1*) | Mm00499303_m1 |
| autophagy related gene 5 (*Atg5*) | Mm01187303_m1 |
| autophagy related gene 7 (*Atg7*) | Mm00512209_m1 |
| sequestosome 1 (*Sqstm1*) | Mm00448091_m1 |
| tumor necrosis factor (*Tnf*) | Mm00443258_m1 |
| interleukin 1 beta (*Il1b*) | Mm00434228_m1 |
| NLR family, pyrin domain containing 3 (*Nlrp3*) | Mm00840904_m1 |
| actin, beta (*Actb*) | Mm02619580_g1 |
| myogenic differentiation 1 (*Myod1*) | Mm00440387_m1 |
| myogenin (*Myog*) | Mm00446194_m1 |
| myostatin (*Mstn*) | Mm01254559_m1 |
| myogenic factor 5 (*Myf5*) | Mm00435125_m1 |
| proliferating cell nuclear antigen (*Pcna*) | Mm00448100_g1 |
| cholinergic receptor, nicotinic, alpha 1 polypeptide (*Chrna1*) | Mm00431629_m1 |
| cholinergic receptor, nicotinic, gamma polypeptide (*Chrng*) | Mm00437419_m1 |
| cholinergic receptor, nicotinic, epsilon polypeptide (*Chrne*) | Mm00437411_m1 |
| histone deacetylase 4 (*Hdac4*) | Mm01299557_m1 |
| histone deacetylase 5 (*Hdac5*) | Mm01246076_m1 |
| myocyte enhancer factor 2C (*Mef2c*) | Mm01340842_m1 |
