# Supplementary material for: Adipose tissue protects against sepsis-induced muscle weakness in mice: from lipolysis to ketones
Source: Crit Care. 2019 Jul 1;23:236. doi: 10.1186/s13054-019-2506-6 (PMC6600878; doi:10.1186/s13054-019-2506-6)
Supplement: Supplementary file 7 — Supplemental Material and Methods. (DOCX 83 kb) [file 13054_2019_2506_MOESM7_ESM.docx]

**Supplemental Material and Methods**

**Animal studies**

The mouse model has been described in detail [1]. In brief, mice were randomly allocated to “sepsis” or “healthy control” groups. Mice in the sepsis groups were anaesthetized after which the central jugular vein was catheterized and connected to a swivel device. This allowed free movement and continuous fluid perfusion. Sepsis was induced by ligation and puncture of the cecum (CLP; 50% ligation of the cecum and single-needle puncture through-and-through). After surgery, animals were transferred to individual cages in a controlled environment (27°C, 12h light/dark). Intravenous fluid resuscitation was started with Plasmalyte A Viaflo (Baxter, Lessines, Belgium) and 6% hydroxyethyl starch in a 4/1 proportion at 0.3 ml/h for the first 20 hours. Six hours post-operatively, mice received subcutaneous antibiotics and analgesia (0.5 mg imipenem and 4.5 µg buprenorphine). Hereafter, antibiotics/analgesia was given every 12 hours until sacrifice (0.5 mg imipenem + 9 µg buprenorphine). From day 1 onwards, septic mice received standard mixed PN (Olimel N7E, Baxter) at 5.8 kcal/day, unless stated otherwise. Mice were excluded if catheter-related problems occurred, such as catheter loss, occlusion, or dislocation. Pain/discomfort was assessed twice daily based on the Mouse Grimace Score [2] and scored as: 0 (no discomfort), 1 (moderate discomfort), 2 (severe discomfort). Cumulative illness scores were calculated to assess the severity of illness. Non-surviving mice received the maximal cumulative score +1. As healthy controls, individually caged healthy mice that were pair-fed to septic mice were used. From day 1 onward, pair-feeding was done by giving mice 1 (study 1-3) or 1.1 gram (study 4) of standard chow twice a day (8 AM and 8 PM), which corresponds in total with 5.8 kcal/day (study 1-3) or 6.55 kcal/day (study 4) respectively. Mice were anesthetized and sacrificed by cardiac puncture at 12 AM on the last day of the experiment.

*Study 1 – Fatty acid mobilization and metabolism in lean and overweight/obese septic mice (n=117)*: 12 week old male C57BL/6JRj mice (#5752053, Janvier SAS, Chassal, France, RRID:MGI:5752053) received standard chow (10% fat, E15745-04, ssniff, Soest, Germany), or a high-fat diet (45% fat, E15744-34, ssniff) for 12 weeks to generate lean and overweight/obese mice. Mice were housed under standard conditions (22°C, 14/10h light/dark) with *ad libitum* access to chow and water. Body weight was quantified weekly. Blood glucose was also monitored weekly and remained comparable between mice on a 10% and 45% fat diet. Only lean animals that had a body weight <35 g and only overweight/obese mice with a body weight ≥35 g but <45 g (to avoid morbid obesity-associated co-morbidities) within the 12 weeks of diet were included. At 24 weeks of age, lean and overweight/obese mice were randomly allocated to “sepsis” or “healthy control”. Animals were sacrificed after 1 or 5 days [day 1: lean healthy control n=15, obese healthy control n=10, lean sepsis n=15, obese sepsis n=15; day 5: lean healthy control n=17, obese healthy control n=15, lean sepsis n=15, obese sepsis n=15].

*Study 2 – Effect of blocking fatty acid mobilization in overweight/obese septic mice on muscle wasting and weakness (n=73)*: B6N.129S-Pnpla2<tm1Eek>/J mice (ATGL-flox; #024278, The Jackson Laboratory, Bar Harbor, ME, USA, RRID:IMSR_JAX:024278) were bred to B6N.FVB.Tg(Adipoq-Cre)1evr/J mice (#010803, The Jackson Laboratory, RRID:IMSR_JAX:010803) to generate adipose tissue-specific ATGL knockout (AAKO) mice. ATGL^flox/flox^ Cre/+ and ATGL^flox/flox^ +/+ mice were mated to generate male ATGL^flox/flox^ Cre/+ (AAKO) and ATGL^flox/flox^ +/+ (wild-type) mice. Genotyping was performed in DNA extracts from tail or ear biopsies. At 12 weeks old, mice were made overweight/obese as described above. At 24 weeks of age, overweight/obese AAKO and wild-type mice were randomly allocated to “sepsis” or “healthy control”. After 5 days, mice were sacrificed [wild-type healthy control n=19, AAKO healthy control n=18, wild-type sepsis n=19, AAKO sepsis n=17].

After sacrifice, suppressed lipolysis in AAKO mice was confirmed by decreased *Pnpla2/Atgl* gene expression in adipose tissue, but not in skeletal muscle or liver, a higher fat mass, lower plasma glycerol concentrations, and less *ex vivo*-released glycerol from adipose tissue compared to wild-type mice (Supplemental Fig. 5). Additionally, in AAKO septic mice, plasma fatty acid concentrations were reduced compared to the wild-type overweight/obese, whereas hepatic markers of fatty acid oxidation were generally lower in AAKO overweight/obese than in wild-type overweight/obese mice (Supplemental Fig. 5).

*Study 3 - Effect of increased lipid availability on muscle wasting and weakness in lean septic mice (n=70)*: Male, 24 week old C57BL/6JRj mice (#5752053, Janvier SAS, RRID:MGI:5752053) were randomly allocated to “sepsis” or “healthy control”. From day 1 onward, septic mice randomly received either standard mixed PN (5.8 kcal/day obtained from: 35% lipids, 49% glucose and 16% amino acids), or a PN consisting of mainly long- and medium-chain triglycerides (5.8 kcal/day obtained from: 90% lipids (Smoflipid® Lipid Injectable Emulsion, Fresenius Kabi, Schelle, Belgium, containing 30% soybean oil, 30% medium-chain triglycerides, 15% fish oil and 25% olive oil) and 10% glucose (glucose50%, Baxter)). After 5 days, mice were sacrificed [healthy control n=24, sepsis receiving PN n=23, sepsis receiving lipid-rich PN n=23].

*Study 4 - Effect of increased ketone body availability on muscle wasting and weakness in lean septic mice (n=49)*: Male, 24 week old C57BL/6JRj mice (#5752053, Janvier SAS, Chassal, France, RRID:MGI:5752053) were randomly assigned to “sepsis” or “healthy control”. From day 1 onward, septic mice received standard mixed PN (5.8 kcal/day) supplemented with twice daily 400 µl subcutaneous injections of either D-glucose (6.25 mg/g/day - 0.75 kcal/day; A3666, Applichem, Darmstadt, Germany) or D,L-beta-hydroxybutyrate sodium salt (5 mg/g/day - 0.75 kcal/day; containing 50% of each isomer, H6501, Sigma-Aldrich, Saint Louis, MO, USA). Mice were sacrificed after 5 days [healthy control n=15, sepsis PN+gluc n=17, sepsis PN+3-hydroxybutyrate n=17]. The bolus injections of 3-HB resulted in transiently increased plasma 3-HB concentrations to levels that were comparable to those observed with extended fasting (2.9 mM after 30 min, 1.1 mM after 60 min, and 0.3 mM after 120 min) [3].

Mice in study 1 were sacrificed after 1 or 5 days, to assess both the acute and prolonged lipolytic response to sepsis (plasma and *ex vivo* glycerol) [4, 5]. Mice in study 2-4 were sacrificed after 5 days, the timeframe required to develop sepsis induced muscle weakness [6], which was the primary endpoint of these studies. For all day 5 experiments, *ex vivo* muscle force measurements served as primary outcomes for sample size calculations. Experiments were continued until 14 individual *ex vivo* muscle force measurements were recorded per group.

***Ex vivo muscle force***

Directly after euthanasia, the EDL muscle was carefully dissected from one hind limb and suspended in a temperature controlled (30°C) organ bath filled with HEPES-fortified Krebs-Ringer solution (10 mM glucose, 5 mM MgCl_2_, 4.5 mM KCl, 120 mM NaCl, 0.7 mM Na_2_HPO_4_ dibasic, 0.9 mM NaH_2_PO_4_ monobasic, 1.2 mM KH_2_PO_4_, 0.57 mM MgSO_4_, 2 mM CaCl_2_, 10 mM HEPES, pH 7.3), continuously gassed with 95% O_2_ and 5% CO_2_. The muscle was mounted vertically between a fixed clamp and a lever-arm for force measurement (300C-LR Dual-Mode muscle lever, Aurora Scientific, Ontario, Canada). The muscle was stimulated by two platinum electrodes, with 1A controlled current pulses (Aurora Scientific). For each muscle, optimal muscle length (L_0_) was determined by the highest produced twitch force. Subsequently, maximal isometric tetanic force was measured by averaging three consecutive tetanic stimuli (180 Hz stimulation frequency, 200 ms duration, 0.2 ms pulse width) with 2 min rest intervals. To calculate the specific maximal isometric tetanic force, the average maximal isometric tetanic force was divided by the calculated muscle cross-sectional area (CSA), determined by dividing the muscle mass by the product of the optimal fiber length (L_F_ = L_0_ x 0.44) and the density of mammalian skeletal muscle (1.06 mg/mm^3^). Data was analyzed with the Dynamic Muscle Analysis (DMA) software from Aurora Scientific.

***Histological analyses***

Cross-sectional paraffin sections of tibialis anterior muscle were stained overnight at 4°C with a monoclonal antibody against PAX7 (1/100, Mab1675, R&D Systems, Minneapolis, MN, USA, RRID:AB_2159833) and subsequently visualized with a HRP-linked secondary antibody (polyclonal goat anti-mouse immunoglobulins/HRP (P0447, Dako, Agilent Technologies, Diegem, Belgium, RRID:AB_2617137)) and 3,3’-diaminobenzidine. The number of PAX7-positive cells per total muscle cross-section was counted and corrected for the surface area of that muscle cross-section. Histological scoring of liver steatosis was performed on hematoxylin and eosin stained liver sections. Lipid accumulation, observed as unstained, clear circles, was scored as: 0 (<5% fat accumulation in 10x field), 1 (5-33% fat accumulation in 10x field), 2 (33-66% fat accumulation in 10x field), or 3 (>66% fat accumulation in 10x field). Inflammatory infiltration was scored as: 0 (no foci per 10x field), 1 (<2 foci per 10x field), 2 (2-4 foci per 10x field), or 3 (>4 foci per 10x field). Presence of ballooning was scored as: 0 (not present) or 1 (some ballooning). Fibrosis was scored as: 0 (not present) or 1 (signs of fibrosis). A summation of the individual scores was made to obtain a global steatosis score. Scoring was performed blinded by two independent evaluators (CG and TD). If scores were divergent, slides were reevaluated together to obtain a consensus.

***Ex vivo glycerol release***

Glycerol release was evaluated in epididymal adipose tissue explants as a measure of lipolysis. Epididymal adipose tissue was cut in small pieces of approximately 3 mm^3^ and incubated in a HEPES-fortified Krebs-Ringer buffer (121 mM NaCl, 4.9 mM KCl, 1.2 mM MgSO_4_, 0.33 mM CaCl, 3.5% defatted BSA, 0.1% glucose, 12 mM HEPES, pH 7.4) at 37°C for 30 minutes. The released glycerol was measured in an aliquot of the buffer with a commercially available kit (Glycerol Assay Kit MAK117, Sigma-Aldrich).

***Gene expression analyses***

Messenger RNA was isolated, and cDNA was quantified in real-time as previously [7]. Commercial TaqMan® assays (Applied Biosystems, Carlsbad, CA, USA) were used for all gene expression analyses (Supplemental table 1). Data were normalized to *Rn18s* or *Hprt* and expressed as fold change of the mean of controls.

***Plasma analyses***

Plasma glucose concentrations were measured in whole blood with a glucose meter after cardiac puncture (Accu-check, Roche, Basel, Switzerland). Plasma glycerol, TNF-α, LDL, HDL, triglycerides, free fatty acids, insulin and 3-hydroxybutyrate were measured using commercially available kits: Glycerol Assay Kit (MAK117, Sigma-Aldrich), Mouse TNF-alpha Quantikine HS ELISA Kit (MHSTA50; R&D Systems), LDL-Cholesterol assay (DZ128A-K; Diazyme Laboratories, Poway, CA, USA), HDL-Cholesterol assay (DZ129A-K; Diazyme), triglyceride quantification kit (Ab65336; Abcam), Free Fatty Acid Fluorometric Assay Kit (7010310; Cayman Chemical Company, Ann Arbor, MI, USA), Insulin Mouse Ultra Sensitive ELISA (90080, Crystal Chem, Downers Grove, IL, USA), EnzyChrom^TM^ Ketone Body Assay Kit (EKBD-100; Bioassay Systems, Hayward, CA, USA).

***Protein expression analyses***

Protein isolation from gastrocnemius muscles was executed as previously described [8]. Immunoblotting was performed with primary antibodies against LC3 (L7543, Sigma-Aldrich, RRID:AB_796155), p62 (H00008878-M01, Novus Biologicals, Littleton, CO, USA, RRID:AB_548364), b-actin (ab3280, Abcam, RRID:AB_303668), ULK1 (D8H5, #8054, Cell Signaling, Danvers, MA, USA, RRID:AB_11178668), phospho-ULK Ser757 (D706U, #14202, Cell Signaling, RRID:AB_2665508), S6K1 (#9202, Cell Signaling, RRID:AB_331676), phosphor-S6K1 Thr389 (#9205, Cell Signaling, RRID:AB_330944), and 4E-BP1 (53H11, #9644, Cell Signaling, RRID:AB_2097841). Secondary horseradish peroxidase-conjugated antibodies (polyclonal goat anti-rabbit immunoglobulins/HRP (P0448, RRID:AB_2617138) and polyclonal goat anti-mouse immunoglobulins/HRP (P0447, RRID:AB_2617137) were purchased from Dako, Agilent Technologies. Blots were visualized with the G:BOX Chemi XRQ (SynGene, Bangalore, India) and analyzed with SynGene software. Data were normalized to b-actin levels and expressed as fold change of the mean of controls.

***Palmitate oxidation***

Oxidation of [1-^14^C]-labelled fatty acids was measured in tissue homogenates as described previously using a HEPES-fortified modified Krebs-Henseleit buffer (mKH; 120.3 mM KCl, 4.8 mM NaCl, 1.2 mM KH_2_PO_4_, 20 mM HEPES-NaOH pH 7.2, 9.5 mM NaHCO_3_, 2.4 mM MgSO_4_) [9]. Fresh skeletal muscle (10% w/v) and liver (5% w/v) biopsies were homogenized in an ice-cold buffer (0.25 M sucrose, 5 mM MOPS-NaOH pH 7.2, 0.1% ethanol) with a Dounce homogenizer. The oxidation of palmitate was assessed in a final volume of 0.5 ml containing 0.1 ml (liver) or 0.2 ml (muscle) homogenate and respectively 0.4 ml or 0.3 ml mKH (final concentrations: 4 mM ATP, 0.5 mM L-carnitine, 50 µM CoA, 0.2 mM DTT, 0.77% defatted BSA (liver) or 0.34% defatted BSA (muscle), 1 mM malate, 0.5 mM ADP, and 0.2 mM [1-^14^C]palmitate (1 µCi/µmol) for liver or 0.1 mM [1-^14^C]palmitate (10 µCi/µmol) for muscle). Incubations were carried out in preheated closed glass vials with a center well containing Whatman paper for CO_2_ trapping, placed in a gyratory water bath at 37°C. The reaction was stopped after 15 min (liver) or 50 min (muscle) by adding 500 µl 6% HClO_4_. Produced [^14^C]CO_2_ and [^14^C]-labelled acid-soluble metabolites (ASM) were determined as before [9]. Oxidation rates are expressed as nmol produced [^14^C]CO_2_ and [^14^C]-labelled ASM per gram wet weight per minute. Under the conditions used, oxidation rates were linear up to 60 minutes in both liver and muscle homogenates, and mainly mitochondrial palmitate oxidation was measured.

**REFERENCES**

1. Derde S, Thiessen S, Goossens C, Dufour T, Van den Berghe G, Langouche L: **Use of a central venous line for fluids, drugs and nutrient administration in a mouse model of critical illness**. *JoVE* 2017, **art.nr. 55553**.

2. Langford DJ, Bailey AL, Chanda ML, Clarke SE, Drummond TE, Echols S, Glick S, Ingrao J, Klassen-Ross T, Lacroix-Fralish ML *et al*: **Coding of facial expressions of pain in the laboratory mouse**. *Nature methods* 2010, **7**(6):447-449.

3. Cahill GF, Jr.: **Fuel metabolism in starvation**. *Annual review of nutrition* 2006, **26**:1-22.

4. Langouche L, Vander Perre S, Thiessen S, Gunst J, Hermans G, D'Hoore A, Kola B, Korbonits M, Van den Berghe G: **Alterations in adipose tissue during critical illness: An adaptive and protective response?** *Am J Respir Crit Care Med* 2010, **182**(4):507-516.

5. Ilias I, Vassiliadi DA, Theodorakopoulou M, Boutati E, Maratou E, Mitrou P, Nikitas N, Apollonatou S, Dimitriadis G, Armaganidis A *et al*: **Adipose tissue lipolysis and circulating lipids in acute and subacute critical illness: effects of shock and treatment**. *Journal of critical care* 2014, **29**(6):1130 e1135-1139.

6. Goossens C, Marques MB, Derde S, Vander Perre S, Dufour T, Thiessen SE, Guiza F, Janssens T, Hermans G, Vanhorebeek I *et al*: **Premorbid obesity, but not nutrition, prevents critical illness-induced muscle wasting and weakness**. *J Cachexia Sarcopenia Muscle* 2017, **8**(1):89-101.

7. Langouche L, Marques MB, Ingels C, Gunst J, Derde S, Vander Perre S, D'Hoore A, Van den Berghe G: **Critical illness induces alternative activation of M2 macrophages in adipose tissue**. *Crit Care* 2011, **15**(5):R245.

8. Langouche L, Vanhorebeek I, Vlasselaers D, Vander Perre S, Wouters PJ, Skogstrand K, Hansen TK, Van den Berghe G: **Intensive insulin therapy protects the endothelium of critically ill patients**. *The Journal of clinical investigation* 2005, **115**(8):2277-2286.

9. Van Veldhoven P, Mannaerts GP: **Comparison of the activities of some peroxisomal and extraperoxisomal lipid-metabolizing enzymes in liver and extrahepatic tissues of the rat**. *The Biochemical journal* 1985, **227**(3):737-741.
